# Structural origins of broadband emission from layered Pb–Br hybrid perovskites†
†Electronic supplementary information (ESI) available. CCDC 1521053–1521055, 1521057–1521060 and 1521067. For ESI and crystallographic data in CIF or other electronic format see DOI: 10.1039/c7sc01590a



**DOI:** 10.1039/c7sc01590a

**Published:** 2017-04-24

**Authors:** Matthew D. Smith, Adam Jaffe, Emma R. Dohner, Aaron M. Lindenberg, Hemamala I. Karunadasa

**Affiliations:** a Department of Chemistry , Stanford University , Stanford , California 94305 , USA . Email: hemamala@stanford.edu; b Department of Materials Science and Engineering , Stanford University , Stanford , California 94305 , USA

## Abstract

Through structural and optical studies of a series of two-dimensional hybrid perovskites, we show that broadband emission upon near-ultraviolet excitation is common to (001) lead-bromide perovskites. Importantly, we find that the relative intensity of the broad emission correlates with increasing out-of-plane distortion of the Pb–(μ-Br)–Pb angle in the inorganic sheets. Temperature- and power-dependent photoluminescence data obtained on a representative (001) perovskite support an intrinsic origin to the broad emission from the bulk material, where photogenerated carriers cause excited-state lattice distortions mediated through electron–lattice coupling. In contrast, most inorganic phosphors contain extrinsic emissive dopants or emissive surface sites. The design rules established here could allow us to systematically optimize white-light emission from layered hybrid perovskites by fine-tuning the bulk crystal structure.

## Abstract 2

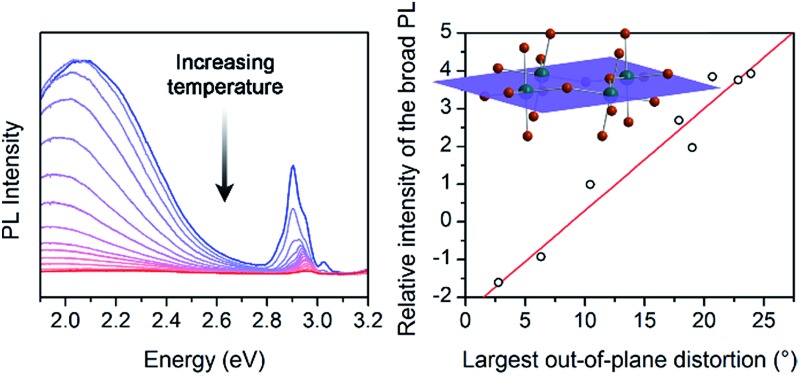
We present synthetic design rules for achieving and optimizing broadband emission from layered halide perovskites.

## Introduction

**Fig. 1:**
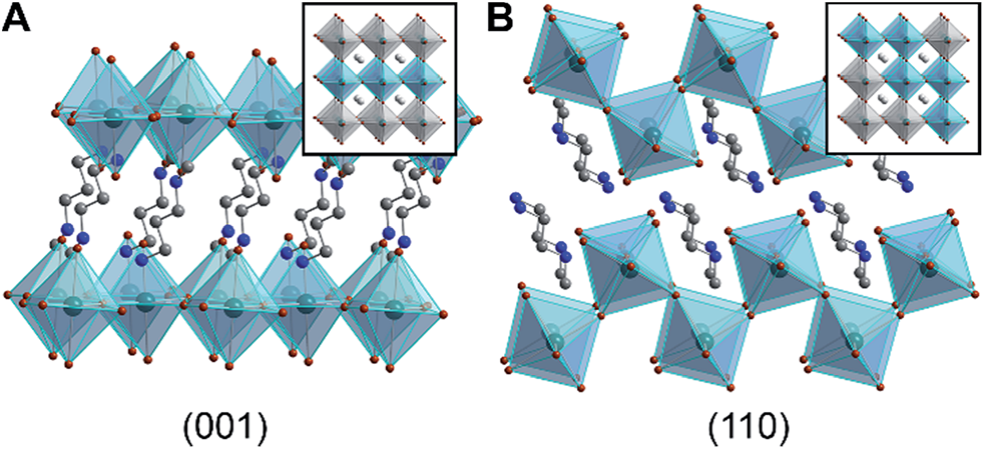
X-ray crystal structures of 2D Pb–Br perovskites, where the inorganic sublattices can be conceptually derived from (A) the (001) and (B) the (110) crystallographic planes of the cubic 3D perovskite structure.^1^ Insets show cubic 3D perovskites with these crystallographic planes highlighted. Green, brown, blue, and gray spheres represent Pb, Br, N, and C atoms, respectively. H and disordered atoms are omitted for clarity.

Broadband white-light emission from a single-phase bulk material without extrinsic emissive sites is unusual. We recently described this phenomenon in two-dimensional (2D) hybrid perovskites.^1,2^ Upon near-ultraviolet excitation, these layered organic–inorganic hybrids (Fig. 1) emit radiation across the entire visible spectrum, approximating sunlight. Most broadband inorganic phosphors contain emissive extrinsic dopants (*e.g.*, Ce^3+^-doped yttrium aluminum garnet)^3,4^ or surface defect sites (*e.g.*, CdSe quantum dots).^5^ Instead, our initial mechanistic studies indicated an intrinsic origin to the broad emission from the bulk perovskites.^2,6^ We proposed that the broad photoluminescence (PL) originated from self-trapped excitons^7,8^—excited electron–hole pairs stabilized through strong coupling to a deformable lattice. Because these “excited-state defects” are mediated through electron–phonon coupling and lattice deformability, which depend on the bulk crystalline structure, we hypothesized that the white-light emission from these materials may be highly amenable to synthetic fine-tuning. Herein, through structural and optical studies on a series of 2D perovskites, we articulate synthetic design rules for realizing and optimizing white-light emission from these hybrid phosphors. Using temperature-dependent photoluminescence studies, we further relate the relative intensity of the broad emission to the thermodynamics of self-trapping.

Broadband phosphors have applications in artificial illumination and displays. Inorganic phosphors are typically synthesized at high temperatures^3^ and require binding agents or resins^4^ for deposition. In contrast, the solution-state film deposition methods available for hybrid perovskites are especially well-suited for inexpensive large-area coatings without the need for additives. Importantly, unlike in purely inorganic solids, hybrid perovskites allow us to systematically tune the inorganic lattice through templating effects from different organic molecules. This allows us to look for correlations between small changes to the bulk crystalline structure and the broadband emission.

Layered hybrid perovskites consist of anionic sheets of corner-sharing metal-halide octahedra partitioned by organic cations (Fig. 1).^9^ The first two examples of Pb–Br perovskite white-light phosphors featured corrugated inorganic sheets.^1,2^ These inorganic sheets can be structurally derived from the cubic three-dimensional (3D) perovskite framework by conceptually slicing along the (110) crystallographic plane^9^ (Fig. 1B). Such (110) perovskites are still rare; the Cambridge Structural Database contains only four (110) Pb–Br perovskites^1,2,10,11^ and a few Sn–I and Pb–I analogs.^2,12,13^ In contrast, (001) Pb–Br perovskites are much more common. In this structure type, the inorganic sheets resemble slices cut along the (001) crystallographic plane of the cubic 3D structure^9^ (Fig. 1A). We find that broadband emission with a large Stokes shift is not unique to the family of (110) Pb–Br perovskites. In fact, all (001) Pb–Br perovskites we have investigated exhibit broad PL features, albeit with a strong dependence on temperature. Although to our knowledge only five white-light-emitting perovskites have been reported to date,^1,2,14,15^ broad PL appears to be much more general to layered perovskites.

At room temperature, typical (001) Pb–Br perovskites exhibit narrow blue/near-UV PL with a small Stokes shift, which has previously been ascribed to radiative emission from free excitons (FEs).^16^ Although excitonic emission in these materials is influenced by material defects and other trap states, the emission remains narrow. Upon cooling, however, we observe the emergence of a new broad PL band with a large Stokes shift, which we attribute to radiative recombination of self-trapped excitons (STEs), analogous to the emission from the (110) Pb–Br perovskites (*N*-MEDA)PbBr_4_ and (EDBE)PbBr_4_ (*N*-MEDA = *N* ^1^-methylethane-1,2-diammonium and EDBE = 2,2′-(ethylenedioxy)bis(ethylammonium)).^1,2^ Self-trapping occurs when charge carriers are stabilized by excited-state lattice distortions induced by the charge carriers themselves.^8^ The evolution of the relative intensities of the narrow and broad PL bands with temperature suggests an equilibrium between “free” and “self-trapped” emissive states, consistent with thermally activated trapping processes.^6^ Importantly, from single-crystal X-ray structures and temperature-dependent PL spectra obtained on a series of (001) Pb–Br perovskites, we see correlations between specific distortions in the bulk inorganic lattice and the intensity of the broad PL relative to that of the narrow emission. These correlations suggest that the broadband emission can indeed be optimized through structural tuning and provide design rules for the synthesis of perovskite white-light emitters. Using these design rules, we synthesize a new (001) Pb–Br perovskite that emits white light with high color rendition at room temperature.

## Results and discussion

### 1. Structural trends in (001) Pb–Br perovskites

The 2D perovskite inorganic sublattice can exhibit local structural distortions in the metal coordination sphere (*i.e.*, deviations from ideal octahedral symmetry)^2,17^ as well as distortions arising from interoctahedral tilting (*i.e.*, deviations of the metal–(μ-halide)–metal angle from 180°).^18^ We examined the X-ray crystal structures of eight (001) Pb–Br perovskites to look for correlations between geometries and optical properties. Crystallographic details of the Pb–Br perovskite structures obtained in this study (seven of which have not been previously reported or required additional refinement) are included in the ESI (Tables S1–S3†) and references to reported structures are given in Table 1.^18–22^

**Table 1:** Largest values for the distortion of selected bond angles in the inorganic sublattices of the 9 Pb–Br perovskites in this study. Here, *D*_tilt_ = 180° – *θ*_tilt_, *D*_out_ = 180° – *θ*_out_, and *D*_in_ = 180° – *θ*_in_, where *θ*_tilt_, *θ*_out_, and *θ*_in_ are shown in Fig. 2

Compound	Reference	Organic cation	*D*_tilt_ (°)	*D*_out_ (°)	*D*_in_ (°)
(BA)_2_PbBr_4_	This work	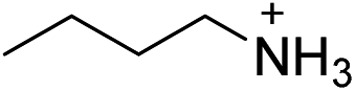	25.2 ± 1.3	2.8 ± 2.8	25.0 ± 0.1
(ETA)_2_PbBr_4_	19	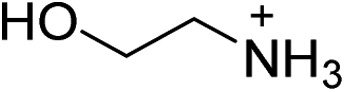	8.1 ± 0.8	6.3 ± 1.6	5.1 ± 1.0
(PEA)_2_PbBr_4_	20	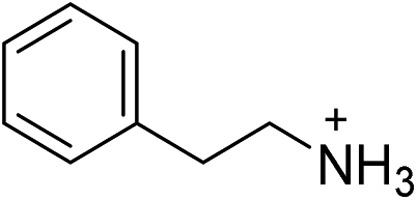	29.2 ± 0.5	10.4 ± 0.5	27.9 ± 0.2
(MPenDA)PbBr_4_	This work,^21^	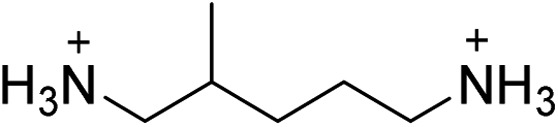	32.6 ± 0.2	17.9 ± 0.4	27.7 ± 0.1
(ODA)PbBr_4_	This work	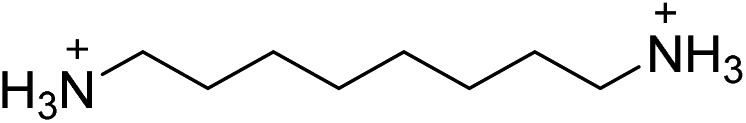	31.8 ± 0.2	19.0 ± 0.4	26.0 ± 0.2
(BDA)PbBr_4_	This work,^22^	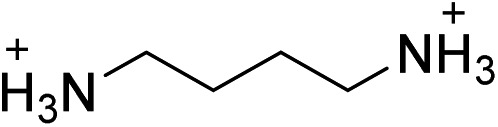	31.6 ± 0.3	20.7 ± 0.7	24.4 ± 0.3
(AEA)PbBr_4_	This work	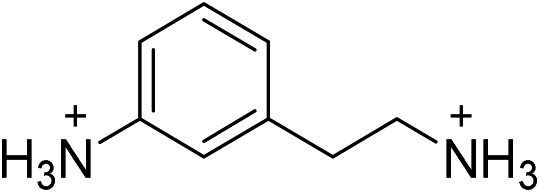	35.7 ± 0.9	22.4 ± 1.9	28.5 ± 0.7
(HIS)PbBr_4_	This work,^18^	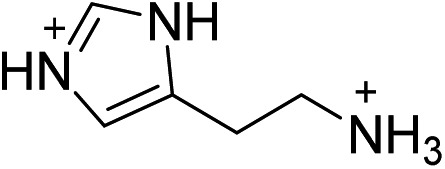	26.1 ± 0.2	22.8 ± 0.2	13.0 ± 0.3
(GABA)_2_PbBr_4_	This work	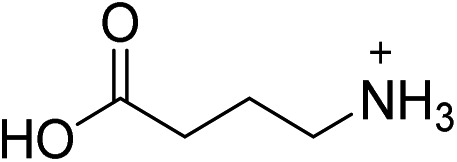	37.8 ± 0.4	23.9 ± 0.9	30.8 ± 0.3

**Fig. 2:**
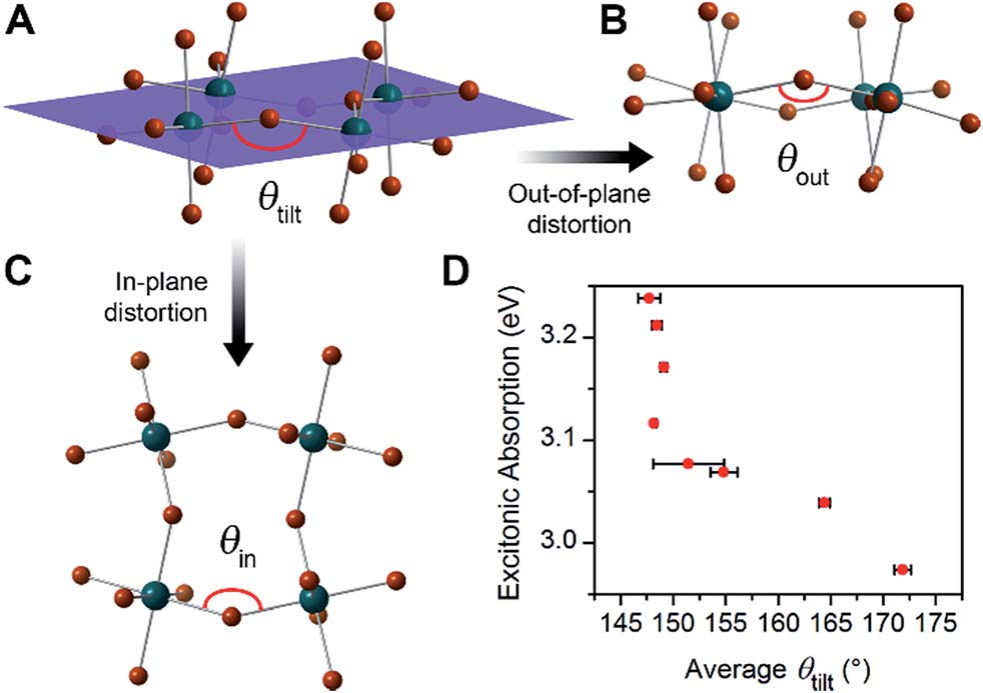
(A) Schematic of interoctahedral tilting (*θ*_tilt_) in a single Pb–Br layer of a (001) perovskite, and its decomposition into (B) out-of-plane (*θ*_out_) and (C) in-plane (*θ*_in_) components. We define in-plane distortion as *D*_in_ = 180° – *θ*_in_ and the out-of-plane distortion as *D*_out_ = 180° – *θ*_out_. (D) Dependence of the excitonic absorption energy at 298 K on *θ*_tilt_ in the series of (001) Pb–Br perovskites. Green and brown spheres represent Pb and Br atoms, respectively.

For the series of (001) Pb–Br perovskite structures discussed here, the average Pb–(μ-Br)–Pb angle (*θ*_tilt_) is 155° with a standard deviation of 9°. This *θ*_tilt_ angle can be separated into two components: in-plane (*θ*_in_) and out-of-plane projections (*θ*_out_) (Fig. 2 and Table 1), where the plane is defined by three adjacent Pb atoms within an inorganic layer. We define in-plane and out-of-plane distortions as the deviation of these angles from linearity, so that in-plane distortion *D*_in_ = 180° – *θ*_in_ and out-of-plane distortion *D*_out_ = 180° – *θ*_out_. The angles *θ*_in_ and *θ*_out_ are not necessarily independent, as previously noted for Sn–I 2D perovskites.^23^ For example, in-plane and out-of-plane distortions can indirectly influence each other through steric and hydrogen-bonding interactions with the organic cations.^23^ We considered a total of 52 structural parameters within the inorganic layers, such as bond distances, bond angles, interoctahedral torsion angles, and deviations from octahedral symmetry within the metal coordination sphere. We discuss the relationship between structure and optical properties in Section 4.

### 2. Optical trends in (001) Pb–Br perovskites: absorption

Layered Pb–Br perovskites are wide-bandgap semiconductors with optical bandgaps of *ca.* 3.4 eV.^24^ Above-bandgap excitation generates excited electrons and holes in the inorganic sheets that are strongly attracted to each other owing to the quantum confinement of the inorganic sheets and to the poor shielding afforded by the low dielectric constant of the adjacent organic layers.^25^ These cooperative effects yield strongly bound electron–hole pairs, or excitons, with large exciton binding energies typically above 300 meV.^24^ The excitonic absorption energies in eight of the perovskites we investigated range from 2.97 to 3.24 eV (Table S5† and Fig. 2D). As average *θ*_tilt_ decreases from 172° to 148°, the excitonic absorption energy increases by *ca.* 270 meV. A similar trend has been reported in 2D Pb–I^26^ and Sn–I^23^ perovskites. Electronic structure calculations on 2D Sn–I perovskites reveal that increases in *D*_tilt_ (increased deviation of *θ*_tilt_ from 180°) reduce the overlap between the metal and halide orbitals, thereby decreasing band dispersion and increasing the bandgap.^23^ Similar distortions in the inorganic framework have been correlated to bandgap increases in the 3D perovskites APbI_3_ (A = CH_3_NH_3_^+^, (H_2_N)_2_CH^+^, and Cs^+^) as well.^27,28^ These trends are also reflected in high-pressure studies of (CH_3_NH_3_)PbI_3_, where increasing interoctahedral tilting with compression increases the bandgap and band-edge PL energy.^29–31^

We then sought to determine if, similar to their optical absorption, the broadband emission from 2D perovskites also showed a dependence on crystal structure. A strong structural dependence would support a significant component of the broad emission being intrinsic to the bulk material, enabling us to tune the emission through synthetic design.

### 3. Optical trends in (001) Pb–Br perovskites: emission

**Fig. 3:**
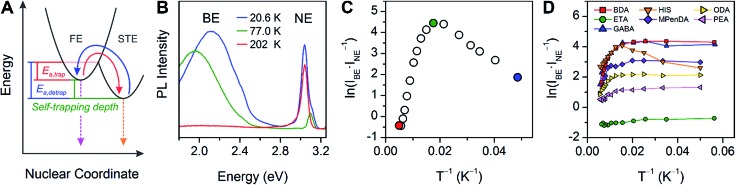
(A) Schematic of exciton self-trapping (red arrow) and detrapping (blue arrow) in 2D Pb–Br perovskites (FE = free exciton state, STE = self-trapped exciton state, *E*_a,trap_ = activation energy for self-trapping, and *E*_a,detrap_ = activation energy for detrapping). FE emission and STE emission are shown in pink and orange, respectively.^6^ (B) Temperature-dependent photoluminescence (PL) of a single crystal of (HIS)PbBr_4_ (HIS = histammonium) showing the narrow emission (NE) at *ca.* 3 eV and the broad emission (BE) at *ca.* 2 eV. (C) Temperature-dependent ratio of integrated BE to NE PL intensity, ln(*I*_BE_·*I*_NE_^–1^), in a single crystal of (HIS)PbBr_4_. The colored symbols in (C) correspond to the spectra shown in (B) depicted in the same color. (D) Temperature-dependent ln(*I*_BE_·*I*_NE_^–1^) ratios for a series of (001) Pb–Br perovskites, abbreviated according to their organic cations (shown in Table 1).

The PL from each (001) Pb–Br perovskite we investigated exhibits similar behavior upon cooling. Although the relative intensities of the broad and narrow emissions are highly variable across our series of eight Pb–Br perovskites, we could always detect the broadband emission at some temperature. To illustrate their emission properties, we first discuss in detail the temperature-dependent PL of (HIS)PbBr_4_ (HIS = histammonium, 4-(2-ammonioethyl)-1*H*-imidazol-3-ium). The room-temperature PL spectrum of a (HIS)PbBr_4_ single crystal shows the narrow FE emission at *ca.* 3 eV that is characteristic of 2D Pb–Br perovskites. With decreasing temperature, we observe the growth of a broad emission at *ca.* 2 eV, with an onset temperature of *ca.* 200 K. This broad emission, which we assign to radiative decay from STEs (Fig. 3A), becomes the dominant PL feature at cryogenic temperatures (Fig. 3B). Because both the FE emission and the STE emission can have some contributions from material defects that affect their energy and/or bandwidth, we refer to the resulting PL bands simply as narrow emission (NE) and broad emission (BE), respectively.

Self-trapping of photogenerated carriers has previously been observed in polar dielectrics such as alkali halides,^32–34^ lead halides,^35,36^ and titania,^37,38^ where strong electron–lattice coupling leads to carrier localization through large excited-state lattice distortions.^8^ Broad PL features, similar to the broad emission we observed from (110) perovskites,^1,2^ have been recently reported at room temperature from films of the (001) perovskite: (C_6_H_11_NH_3_)_2_PbBr_4_.^14,39^ A broad, low intensity red emission in single crystals of CsPbCl_3_ over the temperature range *ca.* 4–150 K has also been attributed to STE emission.^40^

The ratio of integrated intensities of the broad emission (*I*_BE_) to the narrow emission (*I*_NE_) for (HIS)PbBr_4_ evolves as a function of temperature. The plot of ln(*I*_BE_·*I*_NE_^–1^) *vs. T* ^–1^ (Fig. 3C) shows two regimes consistent with a simple model for thermally activated self-trapping, where the FE and STE states are separated by activation-energy barriers for trapping (*E*_a_,_trap_) and detrapping (*E*_a_,_detrap_) (Fig. 3A).^6^ Our recent mechanistic studies on a (110) perovskite are consistent with the presence of several STE states, where permanent material defects can lend further heterogeneity to the excited-state potential-energy surface.^6^ However, for simplicity we show only one STE state in Fig. 3A. At room temperature, we only observe the narrow emission. At these higher temperatures, the lattice has sufficient thermal energy to detrap carriers from STE states back to the FE state. Here, the timescale for carrier detrapping from the STE states back to a FE state (and subsequent radiative FE recombination that results in the narrow emission) is faster than the STE emission pathway. However, upon cooling below 200 K, the BE intensity increases relative to the NE intensity. This suggests that thermal energy is becoming decreasingly sufficient for carriers in the STE state to surmount the activation-energy barrier to detrap back into the FE state (*k*_B_ *T* < *E*_a,detrap_; *k*_B_ = Boltzmann constant). As the temperature is decreased, the absolute intensities of both the narrow and broad emission bands also increase, likely due to reduced nonradiative recombination pathways. The *I*_BE_ : *I*_NE_ ratio reaches its maximum at *ca.* 80 K (green data point at *ca.* 0.0125 K^–1^ in Fig. 3C). With continued cooling below this temperature, *I*_NE_ begins to increase again relative to *I*_BE_. We assign this as a temperature regime in which thermal energy is insufficient to easily exceed the activation-energy barrier for self-trapping (*k*_B_ *T* < *E*_a,trap_), thus hindering FEs from accessing the STE states. Such thermal equilibration between FEs and STEs has also been studied in organic molecular semiconductors such as pyrene.^41,42^ This relationship may be complicated at very low temperatures by processes such as intersystem crossing between singlet and triplet excitons,^24,43^ tunneling^6,44^ between FE and STE states, and other nonlinear processes such as biexcitonic emission.^45^ The continuous evolution of the emission energies of the perovskites suggests that there are no first-order phase transitions at these temperatures.

The difference between energy minima of FE and STE states is the self-trapping depth = *E*_a,detrap_ – *E*_a,trap_ = –Δ*G*_self-trap_ (where Δ*G*_self-trap_ < 0). The ratio of integrated broad and narrow emission intensities (*I*_BE_·*I*_NE_^–1^) at a given temperature is related to Δ*G*_self-trap_ and the radiative emission rates from the STE and FE states (*k*_r,s_ and *k*_r,f_, respectively) through an Arrhenius relation (see ESI† for the full derivation):
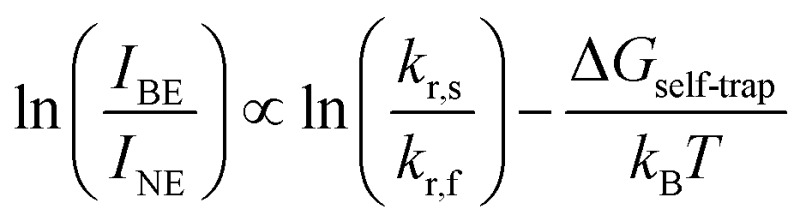
Therefore, we can relate the relative energies of the FE and STE states and the tendency to form STEs to the ratio of PL intensities of the broad and narrow emissions at a given temperature (Fig. 3D).

The broad emission intensity in (HIS)PbBr_4_ at 20.0 K increases in a linear manner (*y* = *y*_0_ + *ax* ^*b*^; where *a* = 4.5(9) × 10^–5^, *b* = 0.96(5), *y*_0_ = 1(2) × 10^–5^) with increasing excitation intensity from 0.371 to 39.9 mW cm^–2^ (Fig. S9†). The intensity of PL from permanent defects typically shows sublinear power dependence with eventual saturation at high excitation intensities owing to the limited number of defects.^46^ The linear power dependence we observe (similar to reported power dependence for excitonic processes in InGaN quantum wells)^47^ and the insensitivity of the emission band shape to excitation power density (Fig. S10†) is consistent, however, with light-generated defects, which should scale with increasing excitation intensity.^2^ We also observe similar broad emission band shape from both single crystals and ball-milled powders of (HIS)PbBr_4_ (Fig. S11†), indicating that the PL does not originate from defects at the crystal/particle surface. We further see the broad emission in perovskite samples crystallized using different methods, suggesting that the emission does not arise solely from lattice defects formed during synthesis. These temperature- and power-dependence studies of emission from crystals of the (001) perovskite (HIS)PbBr_4_ support our assignment of the broad emission originating from STEs, in agreement with our previous work on (110) Pb–Br perovskites.^1,2^

Because the temperature and power dependence of the broad emission in (HIS)PbBr_4_ pointed towards an intrinsic origin, we repeated the temperature-dependent PL measurements using eight Pb–Br perovskites listed in Table 1 (excluding (AEA)PbBr_4_, which is discussed in Section 4). We used ball-milled powders of the perovskites to avoid any angle dependence of incident light or heterogeneity between different crystals. Such heterogeneity in emission energy and lifetime has been previously observed in single crystals of Pb–I perovskites.^48^ The Arrhenius relations for each of the perovskites in our series appear qualitatively similar to that of single-crystal (HIS)PbBr_4_ (Fig. 3D). Upon cooling to *ca.* 80 K, the broad emission increases significantly for all the perovskites except for (ETA)_2_PbBr_4_ and (BA)_2_PbBr_4_, which both exhibit minimal broad emission.

### 4. Structural correlations to the broadband emission in (001) Pb–Br perovskites

We then examined a large number of structural parameters in the room-temperature single-crystal X-ray structures of the eight Pb–Br perovskites, to look for correlations between the emission and the crystal structures. Notably, similar to the excitonic absorption energy in other 2D perovskites,^23,26^ the broad emission also shows a dependence on the distortion along the Pb–(μ-Br)–Pb bond axis, supporting an intrinsic origin from the bulk structure to the emission. Interestingly, however, the key structural parameter that correlates with the broad emission is not *D*_tilt_, but instead its out-of-plane component (*D*_out_).

**Fig. 4:**
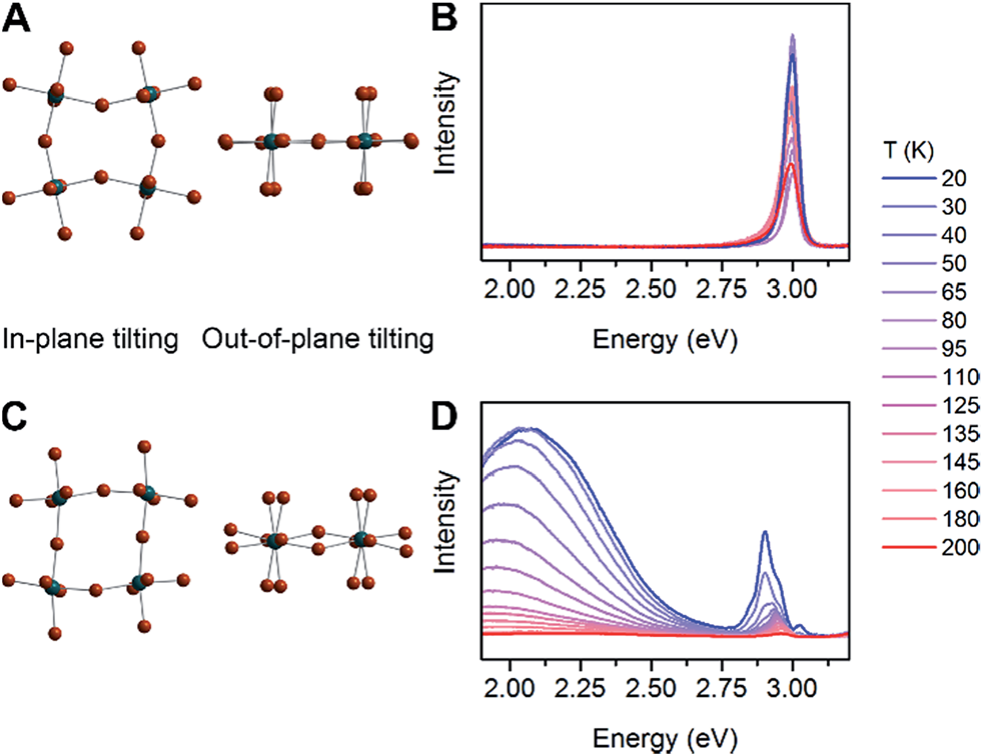
In-plane tilting (*θ*_in_), out-of-plane tilting (*θ*_out_) and variable-temperature photoluminescence (PL) spectra for (A and B) (BA)_2_PbBr_4_ (BA = butylammonium) and (C and D) (HIS)PbBr_4_.

The case for *D*_out_ as the more relevant structural parameter for the broad emission is exemplified by comparing the two (001) perovskites, (BA)_2_PbBr_4_ (BA = butylammonium) and (HIS)PbBr_4_. Fig. 4 shows in-plane and out-of-plane views of the inorganic sheets of these perovskites. Both (BA)_2_PbBr_4_ and (HIS)PbBr_4_ have similar values for the smallest measured *θ*_tilt_ value (highest *D*_tilt_ value) of 155(1)° and 153.9(2)°, respectively. However, in (BA)_2_PbBr_4_ this distortion is almost entirely confined to the plane of the inorganic sheets with smallest measured *θ*_out_ of 177(3)° and *θ*_in_ of 155.0(1)°. This yields a minimal out-of-plane distortion with *D*_out_ = 3(3)° and a large in-plane distortion with *D*_in_ = 25.0(1)° (Fig. 4A). Details of our methodology for angle calculation and error propagation analysis are available in the ESI.† Variable-temperature PL spectra of (BA)_2_PbBr_4_ from 20 to 200 K show mostly the narrow emission with almost no broad emission (Fig. 4B). In contrast, (HIS)PbBr_4_ has a major out-of-plane component and a more minor in-plane component to *θ*_tilt_ with its smallest *θ*_out_ = 157.2(2)° and *θ*_in_ = 167.0(3)°. This affords a large out-of-plane distortion with *D*_out_ = 22.8(2)° and smaller in-plane distortion with *D*_in_ = 13.0(3)° (Fig. 4C). Here, we see a much larger contribution from the broad emission in the variable-temperature PL spectra, with the integrated intensity of the broad emission dominating over that of the narrow emission at temperatures below 200 K (Fig. 4D).

**Fig. 5:**
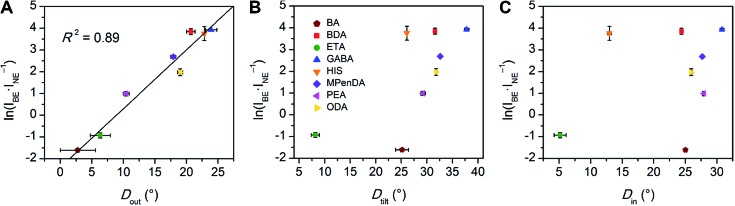
Values of ln(*I*_BE_·*I*_NE_^–1^) for the series of (001) Pb–Br perovskites plotted as a function of (A) largest measured *D*_out_ value, (B) largest measured *D*_tilt_ value, and (C) largest measured *D*_in_ value. The linear fit for (A) is shown to track the correlation between the broad emission and *D*_out_. Error bars correspond to uncertainties arising from structural parameter calculations and from multiple measurements of the ln(*I*_BE_·*I*_NE_^–1^) values (detailed in the ESI†).

We then examined the relationship between *D*_out_ and ln(*I*_BE_·*I*_NE_^–1^) across the series of Pb–Br perovskites. We chose the emission at 80 K for this analysis because ln(*I*_BE_·*I*_NE_^–1^) tends to reach its maximum near this temperature for each perovskite. We find that increasing *D*_out_ correlates linearly with increasing ln(*I*_BE_·*I*_NE_^–1^) across the entire series (Fig. 5A). The strongest correlation is evident when using the largest measured values of *D*_out_ for each perovskite, instead of their average values (Fig. S12†). This is consistent with exciton self-trapping being primarily governed by the local geometry in the inorganic layers. We also observe weak relationships between ln(*I*_BE_·*I*_NE_^–1^) and (i) the distance between terminal Br and Pb atoms and (ii) the torsion angle between terminal Br atoms (Br–Pb···Pb–Br) (Fig. S13A and 13B,† respectively). We note, however, that not all parameters are independent of *D*_out_. For example, *D*_out_ contributes to the terminal Br–Pb···Pb–Br torsion angle and to *D*_tilt_. In contrast to the linear correlation with *D*_out_, no clear trends could be seen for the relationship between ln(*I*_BE_·*I*_NE_^–1^) and *D*_tilt_ or *D*_in_ (Fig. 5B and C). Distortions from octahedral symmetry within the metal coordination sphere also yielded no clear correlations with ln(*I*_BE_·*I*_NE_^–1^) (Fig. S15†). Overall, we tested 52 parameters but observed no clear structural correlation to ln(*I*_BE_·*I*_NE_^–1^), except for *D*_out_.

**Fig. 6:**
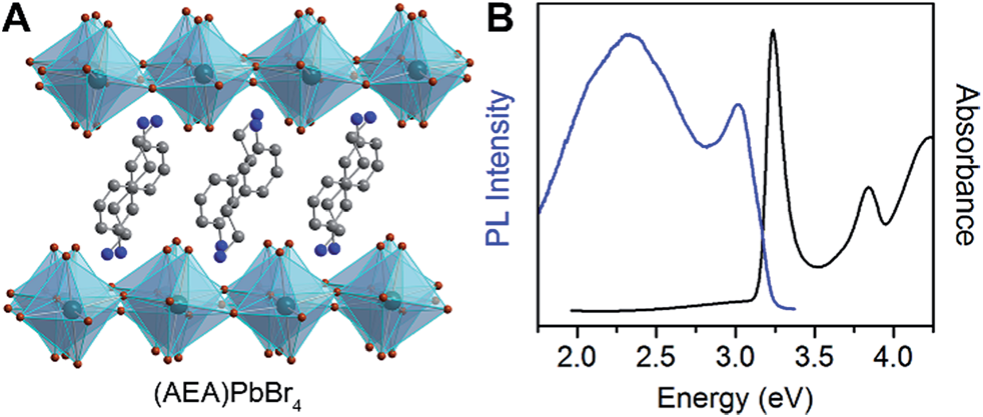
(A) X-ray structure and (B) absorption and emission spectra of (AEA)PbBr_4_ (AEA = 3-(2-ammonioethyl)anilinium), a (001) Pb–Br perovskite with high out-of-plane distortion (*D*_out_) that shows white-light emission at room temperature. Green, brown, blue, and gray spheres represent Pb, Br, N, and C atoms, respectively. H atoms omitted for clarity.

To further test the correlation between *D*_out_ and the relative intensity of the broad emission, we chose the perovskite (AEA)PbBr_4_ (AEA = 3-(2-ammonioethyl)anilinium, Fig. 6A). The inorganic layers of this perovskite are highly distorted, with a high *D*_out_ = 22.4(19)°. We find that (AEA)PbBr_4_ displays higher broad-emission intensity than narrow-emission intensity over the entire temperature range from 20 to 296 K. The room-temperature emission from (AEA)PbBr_4_ (Fig. 6B) has CIE coordinates^3,4^ of (0.29, 0.34) and a color rendering index^3,4^ of 87, corresponding to white light with excellent color rendition.

We are aware of one reported outlier to the structural-optical trends shown here: the (001) Pb–Br perovskite (C_6_H_11_NH_3_)_2_PbBr_4_.^14,39^ This perovskite shows no out-of-plane distortion (*D*_out_ = 0°), yet it exhibits broadband emission at room temperature.^14,39^ Notably, however, this material crystallizes in the polar space group *Cmc*2_1_. Polar materials can exhibit a much higher degree of electron–phonon coupling compared to analogous non-polar materials.^49^ Because exciton self-trapping is mediated by interactions between the exciton and the lattice, electron–phonon coupling should play a significant role in the broad emission. For consistency, we considered only perovskites that crystallize in centrosymmetric space groups for our structural analysis. Examination of polar perovskites may provide additional routes for favoring broadband emission from these tunable materials.

## Conclusions

We show that broadband emission with a large Stokes shift is common to (001) Pb–Br perovskites. The temperature- and power-dependence of the broad emission from a single crystal of a representative (001) Pb–Br perovskite indicates it is intrinsic to the bulk material, consistent with our proposal of self-trapping of photogenerated carriers through excited-state lattice distortions.

Self-trapping has been extensively studied in alkali halides^32–34^ and lead halides,^35,36,50^ which exhibit a number of similarities to lead-halide perovskites, such as band-edge orbital composition. However, unlike in purely inorganic solids, we can induce slight distortions in the inorganic lattice of hybrid perovskites by fine tuning the organic cations. This allows us to systematically study the effects of lattice distortions on their emissive properties. Indeed, our structural and optical studies on a series of eight (001) Pb–Br hybrid perovskites show that ln(*I*_BE_·*I*_NE_^–1^) increases linearly with increasing out-of-plane distortion of the Pb–(μ-Br)–Pb angle (*D*_out_), where *I*_BE_ and *I*_NE_ are the integrated intensities of the broad emission and the narrow emission, respectively.

As we previously noted,^2,6^ we cannot eliminate contributions to the broad emission from permanent material defects where excited charge carriers, including self-trapped carriers, are loosely bound to acceptor or donor defects. Because self-trapping inherently induces local lattice distortions, it is difficult to distinguish between intrinsic self-trapping (mediated purely through electron–lattice coupling) and extrinsic self-trapping (mediated through self-trapped carriers interacting with permanent material defects) by using optical spectra.^8^ Furthermore, the transient lattice defects caused by self-trapping could also lead to permanent lattice defects. For example, self-trapping precedes defect formation in the creation of color centers in alkali-halide crystals.^51^ However, the correlations we see between the perovskites' crystal structures and PL provide compelling evidence that the broad emission has a significant component with an intrinsic, bulk origin.

Emission from most inorganic phosphors arises from extrinsic dopants or from ill-defined surface sites. In contrast, the broad emission from these crystalline hybrid perovskites appears to arise, to a significant degree, from the bulk crystal structure and is amenable to synthetic design. The structural correlations shown here allow us to predict which perovskites will exhibit broadband emission at a given temperature. Furthermore, the modular organic–inorganic architecture of these hybrids allows us to fine-tune the inorganic lattice through organic substitution. Several studies have investigated the templating effects of organic cations on distortions in the inorganic framework.^17,18,52^ Therefore, the design rules established here could allow us to systematically optimize white-light emission from 2D hybrid perovskites.
