# Supplementary material for: Structural origins of broadband emission from layered Pb–Br hybrid perovskites
Source: Chem Sci. 2017 Apr 24;8(6):4497–504. doi: 10.1039/c7sc01590a (PMC5618335; doi:10.1039/c7sc01590a)
Supplement: Supplementary file 1 [file SC-008-C7SC01590A-s001.pdf]

Electronic Supplementary Material (ESI) for Chemical Science.
This journal is © The Royal Society of Chemistry 2017

Supplementary Information for:

Structural origins of broadband emission from layered Pb–Br hybrid perovskites

Matthew D. Smith,[†,§] Adam Jaffe,[†,§] Emma R. Dohner,[†] Aaron M. Lindenberg,[‡] and Hemamala I. Karunadasa[*,†]

Departments of [†]Chemistry and [‡]Materials Science and Engineering, Stanford University, Stanford, California 94305, United States

[§]These authors contributed equally

[*]hemamala@stanford.edu

Experimental details

Table S1–S6

Figures S1–S15

References

Experimental section

All manipulations were conducted in air unless otherwise noted. Solvents were of reagent grade or higher purity. All reagents were purchased from commercial vendors and used as received. Abbreviations used: MPenDA = 2-methyl-1,5-pentanediammonium, BA = *n*-butylammonium, ETA = ethanolammonium or 2-ammonioethanol, PEA = phenethylammonium or 2-phenylethylammonium, ODA = octanediammonium or 1,8-diammoniooctane, BDA = butanediammonium or 1,4-diammoniobutane, GABA = γ-ammoniobutyric acid or 3-carboxypropan-1-ammonium, HIS = histammonium or 4-(2-ammonioethyl)-1H-imidazol-3-ium, AEA = ammonioethylanilinium or 3-(2-ammonioethyl)anilinium.

Perovskite synthesis

In a typical procedure, solid $PbBr_2$ (1 mmol) and the amine (1 mmol for diamines, 2 mmol for monoamines) were combined in 2 mL of 9M HBr and sonicated. Acetone was then added to precipitate a colorless (for perovskites containing BA, BDA, GABA, MPenDA, ODA, PEA) or yellow (for perovskites containing HIS and ETA) crystalline solid. This suspension was further sonicated to homogenize the solid product. The solid was filtered, rinsed with acetone, and dried under reduced pressure. The dried powder was then suspended in toluene or 1,2-dichlorobenzene and ball-milled using a Fritsch Pulverisette 7 planetary ball mill.

Crystallization

Crystals of the Pb–Br perovskites were prepared according to the following typical procedure. Solid $PbBr_2$ (0.34 mmol) and the amine (0.35 mmol for diamines, 0.70 mmol for monoamines) were combined with 3 mL of 9M HBr in a 20 mL scintillation vial. The vial was heated to 100 ˚C for 2 h to dissolve the solid, and then slowly cooled to room temperature at a rate of -2 °C·hr^{-1} to afford crystals suitable for X-ray diffraction or optical measurements. Single crystals were isolated from the mother liquor using Paratone-N oil. The mm-scale crystals used for optical measurements were rinsed with hexanes (3 × 4 mL) to remove the oil and dried under reduced pressure or flowing N_2.

Film deposition

Quartz substrates were cleaned by a 20 minute UV-ozone etch prior to film deposition. Thin films for optical absorption measurements were prepared by spincoating 0.1 M dimethylformamide (DMF) solutions of the corresponding perovskite crystals onto quartz substrates. The films were then annealed at 75 °C for 1 h.

Crystal structure determination

Crystals were coated with Paratone-*N*® oil, mounted on a Kapton® loop, and transferred to a Bruker D8 Venture diffractometer equipped with a Photon 100 CMOS detector or to the Bruker D85 diffractometer at the Advanced Light Source beamline 11.3.1 at the Lawrence Berkeley National Laboratory. Frames were collected using ω and φ scans and unit-cell parameters were refined against all data. The crystals did not show significant decay during data collection. Frames were integrated and corrected for Lorentz and polarization effects using SAINT 8.34a and were corrected for absorption effects using SADABS V2014.[1] Space-group assignments were based upon systematic absences, *E*-statistics, agreement factors for equivalent reflections, and successful refinement of the structures. The structures were solved by direct methods, expanded through successive difference Fourier maps using SHELXS-97 or through the intrinsic phasing method implemented in APEX2.[1,2] Solutions were refined against all data using the SHELXTL-2013[3] software package and OLEX2.[2-5] Data for $(AEA)PbBr_4$ were refined as a non-merohedral twin determined using ROTAX. Hydrogen atoms were inserted at idealized positions and

refined using a riding model with an isotropic thermal parameter 1.2 times that of the attached carbon or nitrogen atom. Thermal parameters for all non-hydrogen atoms were refined anisotropically. In some instances, portions of the organic molecules were refined using a disorder model.

Powder X-ray diffraction (PXRD)

PXRD measurements were performed on a PANalytical X'Pert2 powder diffractometer with a Cu anode ($K\alpha_1$ = 1.54060 Å, $K\alpha_2$ = 1.54443 Å, $K\alpha_2/K\alpha_1$ = 0.50000), a programmable divergence slit with a nickel filter, and a $\mathrm{PIXcel^{1D}}$ detector. Additional PXRD measurements were performed on a Bruker D8 Advance diffractometer equipped with a Cu anode, fixed divergence slits with a nickel filter, and a LYNXEYE detector. The instrument was operated in a Bragg-Brentano geometry with a step size of 0.01° or 0.02° (2θ). Simulated powder patterns were calculated using the crystallographic information files (CIFs) from single-crystal X-ray experiments.

Optical measurements

Variable-temperature static photoluminescence spectra were collected with a spectrograph (Acton Research SpectraPro 500i) equipped with a silicon CCD (Hamamatsu) detector, using excitation from a 375-nm CW diode laser. Samples were cooled using either liquid nitrogen or liquid helium with a Janis ST-500 cryostat. Single-crystal samples were placed on the cold finger with the inorganic layers oriented perpendicular to the incident beam. Powder samples were prepared by mixing a suspension of the ball-milled perovskites in toluene with a solution of poly(methyl methacrylate) (average $M_w \approx 120{,}000$ by GPC) in toluene. This slurry was then allowed to dry at room temperature. For single crystals and powders, the excitation intensity was ca. 0.820 $\mathrm{mW{\cdot}cm^{-2}}$ or 5.74 $\mathrm{mW{\cdot}cm^{-2}}$, respectively, as measured by a Newport 918-UV-L photodiode. For power-dependence measurements on $(HIS)PbBr_4$ single crystals, the spectrograph entrance slits were closed down to maximize spectral resolution (Figure S9). The multiple narrow emission PL peaks have been observed in other layered perovskites, and have been variously ascribed to free and bound excitonic emission[6] or phononic sidebands[7] of the free-excitonic emission. Room-temperature absorption measurements were taken using an Agilent Cary 6000i spectrometer in transmission mode.

Calculation of Pb–Br angles and their error

The distortion values D_{tilt}, D_{out}, and D_{in} were calculated using the Matlab script PbBrAngles_witherror.m, which is available as supporting information. The Matlab script contains all mathematical operations necessary for angle and error calculations and comments are included. Atoms were treated as vectors by using their Cartesian coordinates derived from single-crystal X-ray structures. In order to calculate the in- and out-of-plane components D_{in} and D_{out}, planes were defined by three Pb atoms rather than crystallographic planes because in certain cases, the Pb atoms do not lie in the (001) plane. Calculating projected angles requires an arccosine function. The uncertainty δf in an arbitrary function $f(x)$ is $\delta f = |df/dx|\cdot\delta x$, hence $\delta(\cos^{-1}x) = |(1-x^2)^{-1/2}|\cdot\delta x$. This results in higher error in D values when the θ values are closer to 180°. This does not represent lower precision in a particular X-ray structure, but is a necessary result of correct error propagation.

Structural parameters

In addition to D_{out}, D_{in}, D_{tilt}, and the distance between terminal Br and Pb atoms, we tested many other structural parameters related to the inorganic lattice, which could have potentially yielded a correlation to

$\ln(I_{BE} \cdot I_{NE}^{-1})$. Whenever possible, we calculated and tested largest, smallest, average, and distribution values for each parameter. In total we tested 52 structural parameters in the inorganic lattice. The parameters not shown in the manuscript are as follows (terminal Br atoms are denoted Br_{ax} and bridging Br atoms are denoted Br_{eq}): (1) the set of all unique *cis* intraoctahedral Br–Pb–Br angles including its subsets (2) *cis* Br_{ax}–Pb–Br_{eq} intraoctahedral angles and (3) *cis* Br_{eq}–Pb–Br_{eq} intraoctahedral angles; (4) the set of all unique *trans* intraoctahedral Br–Pb–Br angles, including its subsets (5) *trans* Br_{ax}–Pb–Br_{ax} intraoctahedral angles and (6) *trans* Br_{eq}–Pb–Br_{eq} intraoctahedral angles; (7) all unique intraoctahedral Pb–Br distances, including its subset (8) intraoctahedral distances between Br_{eq} and Pb; intraoctahedral distances between (9) Br_{ax}–Br_{ax}, (10) Br_{ax}–Br_{eq}, and (11) Br_{eq}–Br_{eq}; (12) a measure of octahedral compression (ratio of distance between Br_{ax} atoms to average of *trans* Br_{eq}–Br_{eq} distance), (13) SHAPE (see below), (14) octahedral elongation (λ_{oct}, see below) (15) octahedral angle variance (σ^2_{oct}, see below); (16) the interoctahedral torsion angle between Br_{ax} atoms, and (17) the interoctahedral torsion angle between Br_{eq} atoms.

Calculation of polyhedral distortion

For each crystal structure, Cartesian coordinates were obtained for a lead atom and the six halides that comprise its octahedral coordination environment. These values were supplied to *SHAPE*,[8] a program that calculates continuous shape measures for atomic positions relative to an idealized polyhedron based on minimal distortion paths,[9] generalized interconversion coordinates,[10] and the following algorithm:[11]

$$S = min \frac{\sum_{k=1}^{N} |Q_k - P_k|^2}{\sum_{k=1}^{N} |Q_k - Q_0|^2} \times 100$$

Here, S is a dimensionless continuous symmetry measure obtained by assessing the root-mean-square (rms) deviation of N vertices from their idealized positions. Q_k is a vector containing the coordinates of the N vertices and P_k is the vector for idealized positions. Q_0 is the coordinate vector of the center of mass, and S is normalized by the rms distance from the center of mass to all vertices thus avoiding size effects.

In addition to using S as a measure of polyhedral distortion, two other parameters were calculated, which also provide a measure of polyhedral distortion: octahedral elongation (λ_{oct}) and octahedral angle variance (σ^2_{oct}). Here, λ_{oct} describes the deviation of an octahedron's six Pb–Br bond distances away from the Pb–Br bond distance of a regular octahedron with the same volume. Similarly, σ^2_{oct} describes deviations of the twelve *cis* Br–Pb–Br angles within an octahedron away from 90°. These parameters are calculated as follows:[12]

$$\lambda_{oct} = \frac{1}{6}\sum_{i=1}^{6}\left(\frac{d_i}{d_0}\right)^2$$

$$\sigma^2{}_{oct} = \frac{1}{11}\sum_{i=1}^{12}(\alpha_i - 90)^2$$

Here, d_i is the Pb–Br bond length, d_0 is the Pb–Br bond length in a regular octahedron of the same volume, and α_i is the Br–Pb–Br angle within the octahedron. Note that in calculating σ^2_{oct}, Bessel's correction was used to reduce error in population variance due to a finite sample count, hence a prefactor

of 1/(n – 1) was employed where n is the number of unique Br–Pb–Br angles. This gives a prefactor of 1/11 instead of 1/12.

Calculation of the Arrhenius relation and self-trapping depth. Following photoexcitation, we assume that the free-exciton (FE) and self-trapped-exciton (STE) states are related by the configuration coordinate diagram shown in the figure. We can use Arrhenius relations to relate the self-trapping depth ($-\Delta G_{self-trap}$; given by the difference in activation energies for trapping and detrapping so that $\Delta G_{self-trap} = E_{a,trap} - E_{a,detrap} < 0$) with the relative integrated intensity of the FE and STE emissions, as determined by the following derivation.

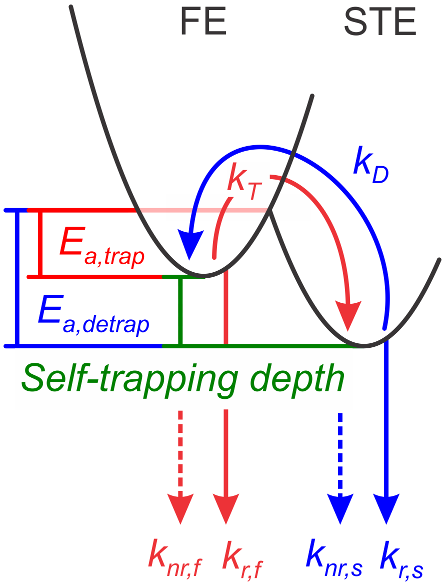

$$\frac{dN_f(t)}{dt} = -N_f\left(k_{r,f} + k_{nr,f} + k_T\right) + k_D N_s = -k_f N_f + k_D N_s$$
$$\frac{dN_s(t)}{dt} = -N_s\left(k_{r,s} + k_{nr,s} + k_D\right) + k_T N_f = -k_s N_s + k_T N_f$$

where $N_f(t)$ and $N_s(t)$ are the populations of the FE and STE states as a function of time, respectively, and $k_{r,f}$, $k_{nr,f}$, $k_{r,s}$, $k_{nr,s}$, k_T, and k_D are the rate constants associated with radiative emission from the FE state, non-radiative decay from the FE state, radiative emission from the STE state, non-radiative decay from the STE state, trapping from the FE to the STE state, and detrapping from the STE to FE state, respectively. We define $k_f = k_{r,f} + k_{nr,f} + k_T$ and $k_s = k_{r,s} + k_{nr,s} + k_D$ for simplicity.

Solving the system of equations yields:

$$N_f(t) = N_0 e^{-\frac{t}{2}(k_f + k_s)}\left[cosh\frac{t}{2}\sqrt{\gamma} + \frac{\left(k_s - k_f\right)}{\sqrt{\gamma}} sinh\frac{t}{2}\sqrt{\gamma}\right]$$

and

$$N_s(t) = \left(\frac{2N_0 k_T}{\sqrt{\gamma}}\right) e^{-\frac{t}{2}(k_f + k_s)} sinh\frac{t}{2}\sqrt{\gamma}$$

where $\gamma = \left(k_f - k_s\right)^2 + 4k_D k_T$

For steady-state (continuous-wave, time-integrated) PL experiments, a general expression for PL intensity[13] is:

$$I \propto k_r \int_0^\infty N(t)dt$$

Evaluating the intensities for the free and self-trapped states, we find:

$$I_{FE} \propto \frac{k_{r,f} N_0 k_s}{k_f k_s - k_D k_T}$$

and

$$I_{STE} \propto \frac{k_{r,s} N_0 k_T}{k_f k_s - k_D k_T}$$

We then obtain a general expression for the ratio of steady-state intensities for a two-state system:

$$\frac{I_{FE}}{I_{STE}} \propto \frac{k_{r,f}k_s}{k_{r,s}k_T} = \frac{k_{r,f}\left(k_{r,s} + k_{nr,s} + k_D\right)}{k_{r,s}k_T}$$

Assuming that exciton trapping and detrapping are primarily thermally activated processes, the following Arrhenius relations hold for a given temperature:

$$k_T = Ae^{-\frac{E_{a,trap}}{k_B T}}$$

$$k_D = Be^{-\frac{E_{a,detrap}}{k_B T}}$$

where $E_{a,trap}$ and $E_{a,detrap}$ are the activation energies for exciton trapping and detrapping, respectively, k_B is Boltzmann's constant, and A and B are exponential prefactors, which we assume are temperature-independent over the range studied.

Therefore,

$$\frac{k_T}{k_D} = \frac{A}{B}e^{-\frac{\left(E_{a,trap} - E_{a,detrap}\right)}{k_B T}} = \frac{A}{B}e^{-\frac{\left(\Delta G_{self-trap}\right)}{k_B T}}$$

Substituting into the expression for the ratio of intensities, we obtain:

$$\frac{I_{FE}}{I_{STE}} \propto \frac{k_{r,f}\left(k_{r,s} + k_{nr,s} + \frac{B}{A}k_T e^{\frac{\Delta G_{self-trap}}{k_B T}}\right)}{k_{r,s}k_T}$$

If we assume that trapping is much faster than radiative and non-radiative decays from the STE state, i.e., that $k_{r,s} + k_{nr,s} \ll k_T$, we can make the following approximation:

$$\frac{I_{FE}}{I_{STE}} \propto \frac{k_{r,f}}{k_{r,s}}e^{\frac{\Delta G_{self-trap}}{k_B T}}$$

Or alternatively,

$$\frac{I_{STE}}{I_{FE}} \propto \frac{k_{r,s}}{k_{r,f}}e^{-\frac{\Delta G_{self-trap}}{k_B T}}$$

Below 80 K, the approximation $k_{r,s} + k_{nr,s} \ll k_T$ may no longer hold, since carrier self-trapping may become decreasingly likely.

Finally, we make the following approximation:

$$\frac{I_{STE}}{I_{FE}} \approx \frac{I_{BE}}{I_{NE}}$$

where I_{BE} and I_{NE} are the intensities for the broad and narrow emission processes. We attribute the narrow and broad emissions to free and self-trapped exciton states, respectively, however they may also have some contributions from material defects.

Thus,

$$\frac{I_{BE}(T)}{I_{NE}(T)} \propto \frac{k_{r,s}}{k_{r,f}}e^{-\frac{\Delta G_{self-trap}}{k_B T}}$$

Table S1. Crystallographic data[a] for $(MPenDA)PbBr_4$, $(BA)_2PbBr_4$, and $(ODA)PbBr_4$

	$(MPenDA)PbBr_4$	$(BA)_2PbBr_4$	$(ODA)PbBr_4$
Empirical Formula	$C_6H_{18}N_2PbBr_4$	$C_8H_{24}N_2PbBr_4$	$C_8H_{22}N_2PbBr_4$
Formula Weight, $g\cdot mol^{-1}$	645.04	675.12	673.10
Temperature, K	298(2)	298(2)	298(2)
Crystal System	Monoclinic	Orthorhombic	Monoclinic
Space group	$C2/c$	$Pbca$	$P2_1/c$
a, Å	24.4632(15)	8.3343(3)	13.807(3)
b, Å	8.0039(4)	8.2225(4)	7.9891(14)
c, Å	8.1963(5)	27.6171(15)	8.2769(14)
β, °	99.745(2)	90	104.340(6)
Volume, $Å^3$	1581.69(16)	1892.57(15)	884.5(3)
Z	4	4	2
Density (calculated), $g\cdot cm^{-3}$	2.705	2.369	2.527
Absorption coefficient, mm^{-1}	20.742	17.341	18.551
$F(000)$	1156.0	1232.0	612.0
Crystal size, mm^3	0.01 × 0.01 × 0.1	0.1 × 0.1 × 0.01	0.05 × 0.03 × 0.01
θ range, °	2.682 to 26.422	2.855 to 27.576	2.970 to 30.49
Index ranges	$-30 \le h \le 30$<br>$-9 \le k \le 9$<br>$-10 \le l \le 10$	$-10 \le h \le 9$<br>$-9 \le k \le 9$<br>$-33 \le l \le 33$	$-18 \le h \le 19$<br>$-11 \le k \le 11$<br>$-10 \le l \le 11$
Reflections collected/unique	33452/1548	28686/1717	12418/2706
Completeness to θ_{max}	0.996	1	1
Max. and min. transmission	0.430, 0.240	0.383, 0.276	0.746, 0.363
Data/restraints/parameters	1548/77/87	1717/24/72	2706/68/90
Goodness-of-fit on F^2	1.093	1.125	1.046
Final R indices $[I > 2\sigma(I)]$[b]	$R_1 = 0.0199$<br>$wR_2 = 0.0430$	$R_1 = 0.0276$<br>$wR_2 = 0.0602$	$R_1 = 0.0272$<br>$wR_2 = 0.0694$
R indices (all data)[b]	$R_1 = 0.0239$<br>$wR_2 = 0.0446$	$R_1 = 0.0436$<br>$wR_2 = 0.0658$	$R_1 = 0.0373$<br>$wR_2 = 0.0732$
Largest diff. peak and hole, $e\cdot Å^{-3}$	0.969, –0.724	0.855, –0.619	1.442, –0.754

[a]Obtained with monochromated Mo Kα (λ = 0.71073 Å) radiation
[b]$R_1 = \Sigma||F_o| - |F_c||/\Sigma|F_o|$, $wR_2 = [\Sigma w(F_o^2 - F_c^2)^2/\Sigma(F_o^2)^2]^{1/2}$

Table S2. Crystallographic data for $(GABA)_2PbBr_4$[a], $(AEA)PbBr_4$[b], and $(BDA)PbBr_4$[a]

	$(GABA)_2PbBr_4$	$(AEA)PbBr_4$	$(BDA)PbBr_4$
Empirical Formula	$C_8H_{20}O_4N_2PbBr_4$	$C_8H_{14}N_2PbBr_4$	$C_4N_2H_{14}PbBr_4$
Formula Weight, g·mol^{-1}	2205.18	665.01	616.97
Temperature, K	298(2)	298(2)	298(2)
Crystal System	Monoclinic	Monoclinic	Triclinic
Space group	$P2_1/c$	$P2_1/c$	$P\bar{1}$
a, Å	24.7721(12)	7.7688(3)	8.0135(4)
b, Å	8.0709(4)	8.5108(4)	8.4270(4)
c, Å	56.964(3)	24.6314(11)	10.7367(5)
α, β, γ, °	90, 97.137(2), 90	90, 95.197(2), 90	78.957(2),<br>69.565(2),<br>89.542(1)
Volume, Å^3	11300.6(10)	1621.90(12)	665.47(6)
Z	8	4	2
Density (calculated), g·cm^{-3}	2.592	2.724	3.079
Absorption coefficient, mm^{-1}	17.452	24.973	24.642
F(000)	8064.0	1192.0	548.0
Crystal size, mm^3	0.08 × 0.04 × 0.01	0.1 × 0.1 × 0.01	0.1 × 0.08 × 0.06
θ range, °	2.409 to 25.815	2.76 to 31.52	2.468 to 28.277
Index ranges	$-30 \le h \le 30$<br>$-9 \le k \le 9$<br>$-70 \le l \le 70$	$-9 \le h \le 9$<br>$-10 \le k \le 10$<br>$-29 \le l \le 29$	$-10 \le h \le 10$<br>$-11 \le k \le 11$<br>$-14 \le l \le 14$
Reflections collected/unique	205643/22216	32430/3017	6236/6236
Completeness to θ_{max}	0.999	1.016 (twin)	1.887 (twin)
Max. and min. transmission	0.4296, 0.2782	0.300, 0.092	0.431, 0.199
Data/restraints/parameters	22216/0/1053	3017/83/140	6236/40/106
Goodness-of-fit on F^2	1.013	1.122	1.036
Final *R* indices $[I > 2\sigma(I)]$[c]	$R_1 = 0.0493$<br>$wR_2 = 0.0757$	$R_1 = 0.0710$<br>$wR_2 = 0.2032$	$R_1 = 0.0378$<br>$wR_2 = 0.0712$
R indices (all data)[c]	$R_1 = 0.1251$<br>$wR_2 = 0.0928$	$R_1 = 0.0780$<br>$wR_2 = 0.2092$	$R_1 = 0.0638$<br>$wR_2 = 0.0790$
Largest diff. peak and hole, e·Å^{-3}	1.907, –1.615	4.194, –2.671	1.304, –1.236

[a]Obtained with monochromated Mo Kα ($\lambda = 0.71073$ Å) radiation
[b]Obtained with synchrotron ($\lambda = 0.7749$ Å) radiation
[c]$R_1 = \Sigma||F_o| - |F_c||/\Sigma|F_o|$, $wR_2 = [\Sigma w(F_o^2 - F_c^2)^2/\Sigma(F_o^2)^2]^{1/2}$

Table S3. Crystallographic data[a] for (HIS)$PbBr_4$ at 100 and 298 K

	100 K	298 K
Empirical Formula	$C_5H_{11}N_3PbBr_4$	$C_5H_{11}N_3PbBr_4$
Formula Weight, g·mol^{-1}	639.97	639.97
Temperature, K	100(2)	298(2)
Crystal System	Monoclinic	Monoclinic
Space group	$P2_1/c$	$P2_1/c$
a, Å	10.5412(13)	10.6055(13)
b, Å	11.5023(14)	11.6091(14)
c, Å	11.9250(14)	11.9312(14)
β, °	109.996(3)	110.061(3)
Volume, Å^3	1358.7(3)	1379.8(3)
Z	4	4
Density (calculated), g·cm^{-3}	3.129	3.081
Absorption coefficient, mm^{-1}	24.147	23.777
F(000)	1136.0	1136.0
Crystal size, mm^3	0.13 × 0.1 × 0.02	0.13 × 0.1 × 0.02
θ range, °	2.538 to 35.091	2.526 to 32.760
Index ranges	$-16 \le h \le 16$<br>$-17 \le k \le 17$<br>$-18 \le l \le 18$	$-15 \le h \le 15$<br>$-16 \le k \le 16$<br>$-17 \le l \le 17$
Reflections collected/unique	44328/5184	31826/4219
Completeness to θ_{max}	0.999	1
Max. and min. transmission	0.343, 0.136	0.340, 0.137
Data/restraints/parameters	5184/0/119	4219/0/119
Goodness-of-fit on F^2	1.067	1.065
Final *R* indices [$I > 2\sigma(I)$][b]	$R_1 = 0.0177$<br>$wR_2 = 0.0394$	$R_1 = 0.0230$<br>$wR_2 = 0.0540$
R indices (all data)[b]	$R_1 = 0.0222$<br>$wR_2 = 0.0407$	$R_1 = 0.0292$<br>$wR_2 = 0.0562$
Largest diff. peak and hole, e·Å^{-3}	1.610, –1.231	1.390, –1.561

[a]Obtained with monochromated Mo K*α* ($\lambda = 0.71073$ Å) radiation

[b]$R_1 = \Sigma||F_o| - |F_c||/\Sigma|F_o|$, $wR_2 = [\Sigma w(F_o^2 - F_c^2)^2/\Sigma(F_o^2)^2]^{1/2}$

Table S4. Continuous symmetry measures (*S*) associated with distortions in the lead-bromide octahedra for the (001) perovskites. Higher *S* values denote greater distortion from ideal octahedral geometry. For perovskites with multiple unique Pb–Br octahedra, average *S* values are given with standard deviations. The overall average value is 0.187 with a standard deviation of 0.173.

Perovskite	*S* (dimensionless)
$(MPenDA)PbBr_4$	0.036
$(BA)_2PbBr_4$	0.133
$(ETA)_2PbBr_4$	0.088
$(PEA)_2PbBr_4$	0.148(1)
$(ODA)PbBr_4$	0.050
$(BDA)PbBr_4$	0.106(3)
$(GABA)_2PbBr_4$	0.09(2)
$(HIS)PbBr_4$	0.645
$(AEA)PbBr_4$	0.329

Table S5. Excitonic absorption energies for the perovskites measured at room temperature

Perovskite	Energy (eV)
$(BA)_2PbBr_4$	3.07
$(ETA)_2PbBr_4$	2.97
$(PEA)_2PbBr_4$	3.08
$(ODA)PbBr_4$	3.12
$(BDA)PbBr_4$	3.17
$(GABA)_2PbBr_4$	3.21
$(HIS)PbBr_4$	3.04
$(AEA)PbBr_4$	3.24

Table S6. Bond angles in the inorganic lattices of the 4 Pb–Br perovskites with multiple unique values. Here, $D_{tilt} = 180^{\circ} - \theta_{tilt}$, $D_{out} = 180^{\circ} - \theta_{out}$, and $D_{in} = 180^{\circ} - \theta_{in}$.

Perovskite	D_{in} (°)	D_{out} (°)	D_{tilt} (°)
$(GABA)_2PbBr_4$	18.9(5)	15(1)	23.8(5)
	19.9(5)	16(1)	25.4(6)
	23.5(4)	16(1)	28.3(6)
	22.1(3)	17.8(8)	28.1(4)
	21.2(5)	20(1)	28.6(5)
	26.2(3)	19.8(8)	32.4(4)
	28.7(3)	20.6(8)	34.8(4)
	30.8(3)	21.6(9)	37.1(4)
	28.2(3)	22.1(9)	35.3(4)
	23.1(4)	22.7(8)	32.0(4)
	26.9(4)	23.3(9)	35.1(4)
	30.1(3)	23.9(9)	37.8(4)
$(BDA)PbBr_4$	23.7(3)	19.2(7)	30.2(3)
	24.4(3)	20.7(2)	31.6(3)
$(HIS)PbBr_4$	3(2)	4(1)	5.1(9)
	13.0(3)	22.8(2)	26.1(2)
$(PEA)_2PbBr_4$	27.8(2)	1(6)	28(7)
	27.9(2)	1(5)	28(6)
	27.4(2)	10.4(5)	29.1(5)
	27.5(2)	10.4(5)	29.2(5)

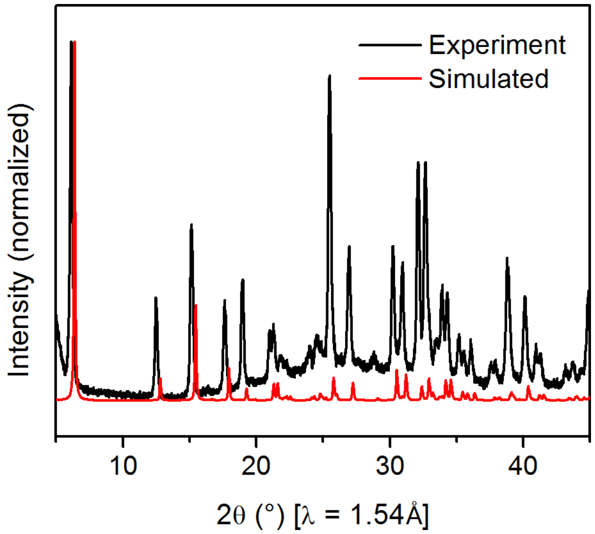

Figure S1. Powder XRD pattern of $(BA)_2PbBr_4$.

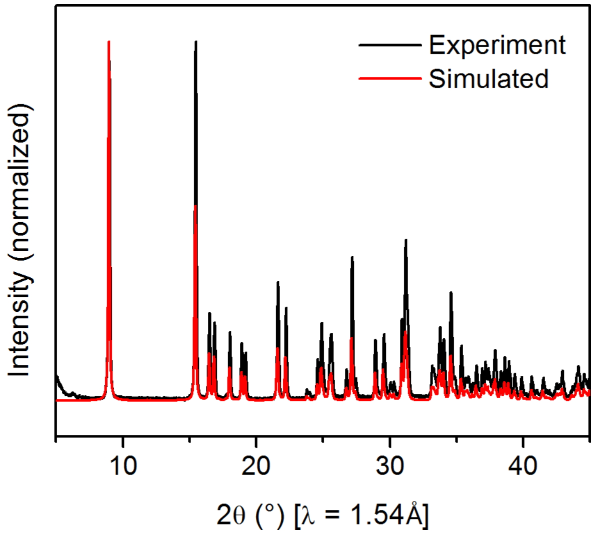

Figure S2. Powder XRD pattern of $(BDA)PbBr_4$.

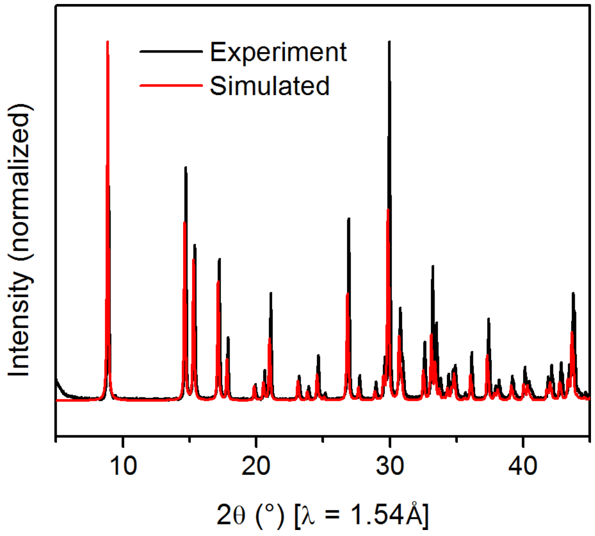

Figure S3. Powder XRD pattern of $(ETA)_2PbBr_4$.

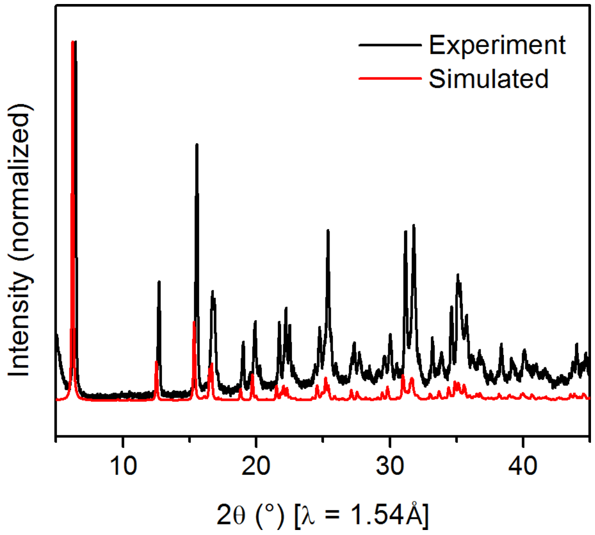

Figure S4. Powder XRD pattern of $(GABA)_2PbBr_4$.

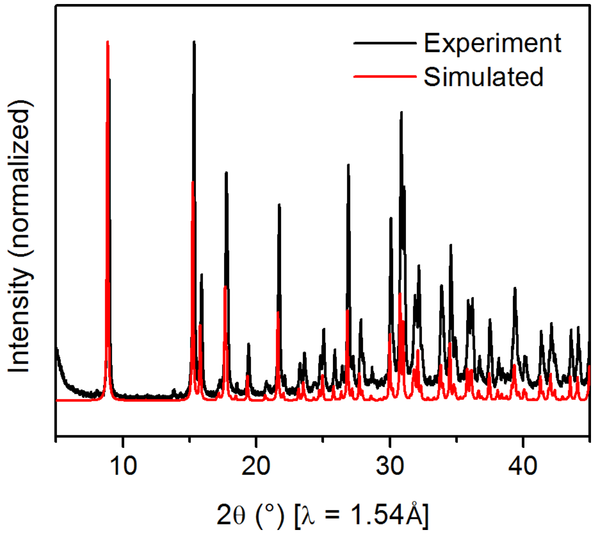

Figure S5. Powder XRD pattern of (HIS)$PbBr_4$.

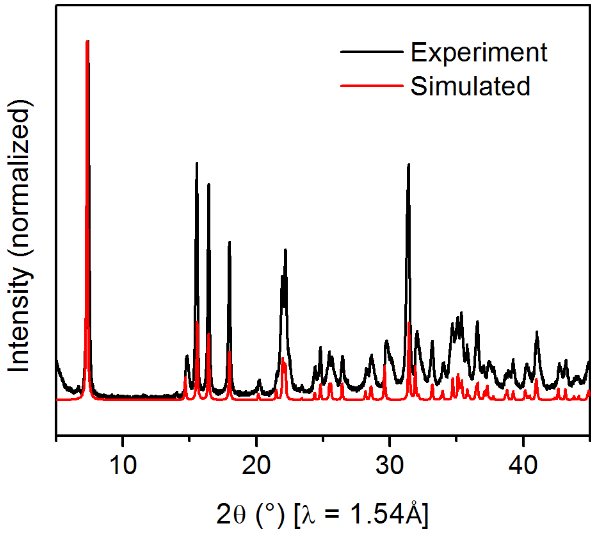

Figure S6. Powder XRD pattern of (MPenDA)$PbBr_4$.

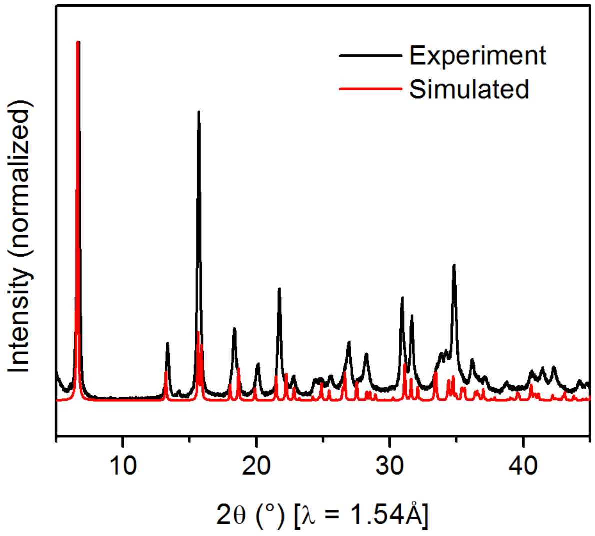

Figure S7. Powder XRD pattern of (ODA)$PbBr_4$.

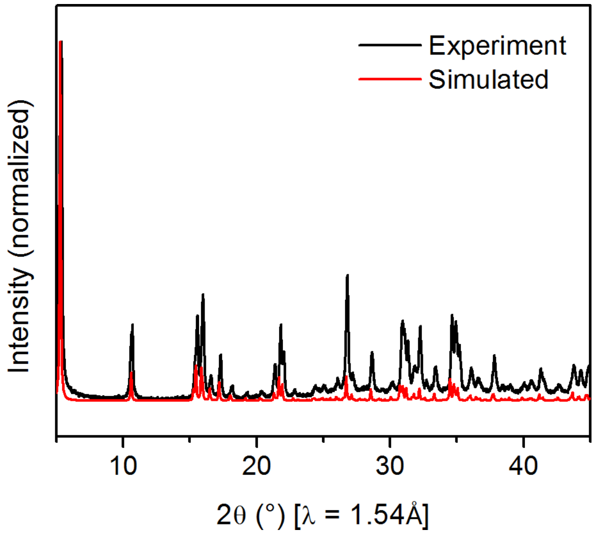

Figure S8. Powder XRD pattern of $(PEA)_2PbBr_4$.

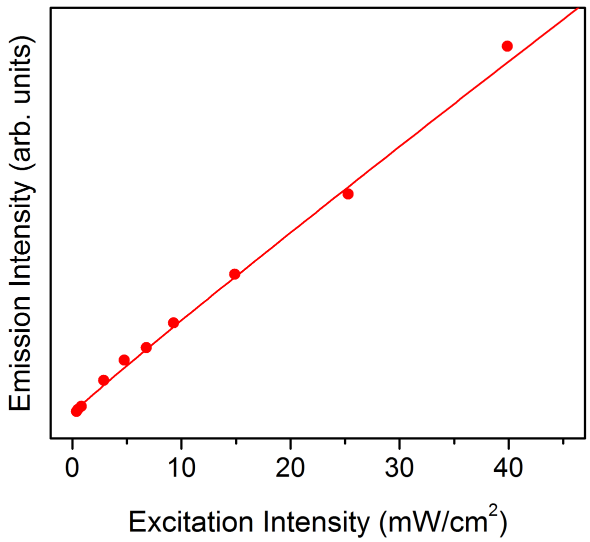

Figure S9. Relationship between excitation and emission intensities for (HIS)$PbBr_4$, showing linear dependence for the broad emission (BE) ($y = y_0 + ax^b$; where $a = 4.5(9) \times 10^{-5}$, $b = 0.96(5)$, $y_0 = 1(2) \times 10^{-5}$) at 20.0 K.

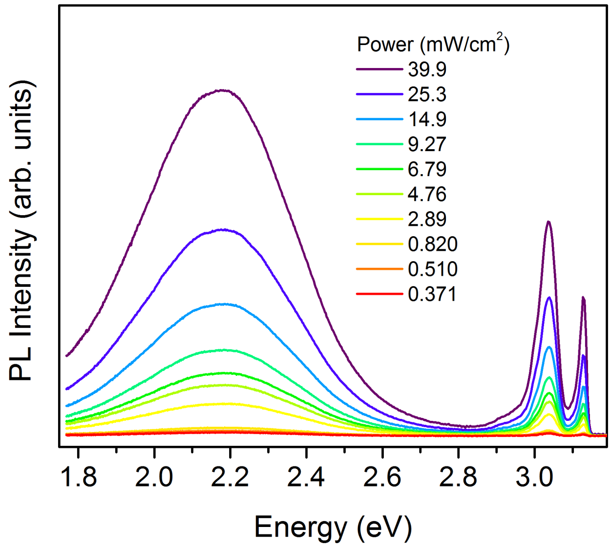

Figure S10. Emission profiles at varying excitation intensities for a crystal of (HIS)$PbBr_4$ at 20.0 K.

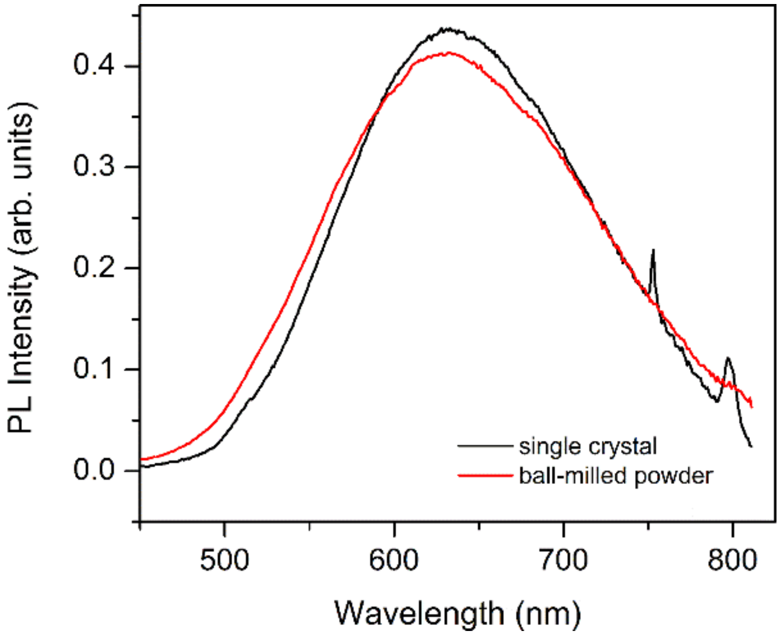

Figure S11. Photoluminescence (PL) from a single crystal and ball-milled powder of $(HIS)PbBr_4$. Ball-milled powder has a particle size of ca. 1 μm and the single crystal has dimensions of 100 × 100 × 25 μm. The peaks in the single-crystal spectrum at 750 and 850 nm are from higher harmonics of the laser excitation source.

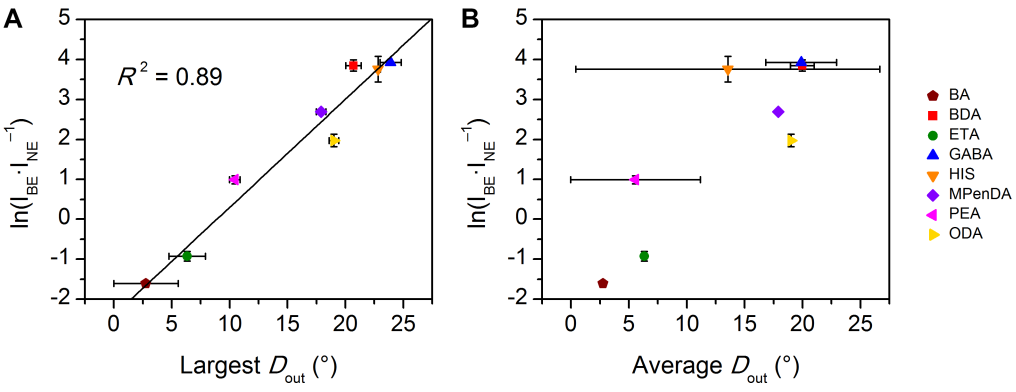

Figure S12. Values of $\ln(I_{BE} \cdot I_{NE}^{-1})$ for the series of (001) Pb–Br perovskites plotted as a function of (A) largest measured D_{out} value and (B) average measured D_{out} value. Importantly, the horizontal error bars in (B) correspond to standard deviations in D_{out} values for the four perovskites with multiple unique values (Table S6). Note that the correlation is much stronger when considering the largest values of D_{out}, which is consistent with self-trapping being related to local distortions in the inorganic layers.

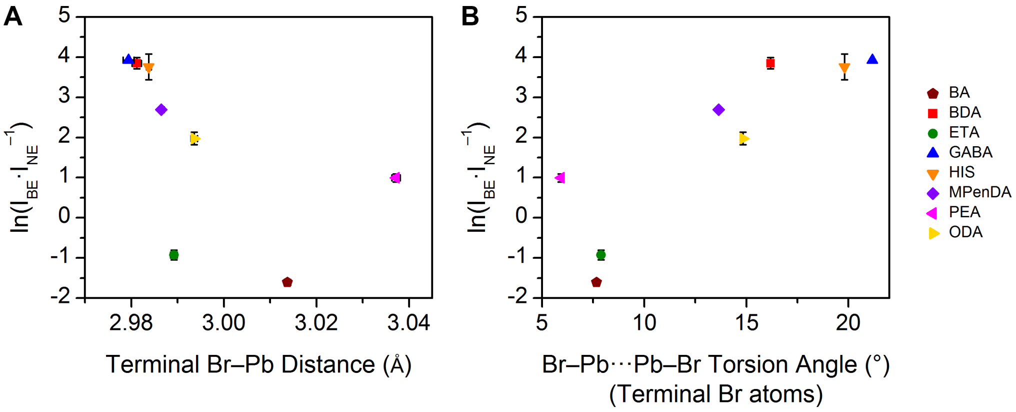

Figure S13. Values of $\ln(I_{BE} \cdot I_{NE}^{-1})$ plotted as a function of (A) the distance between Pb and terminal Br atoms and (B) the torsion angle between terminal Br atoms of adjacent Pb–Br octahedra. Both show weak correlation, although the Pb–Br distance in (A) shows significant outliers and the torsion angle in (B) is not independent of D_{out}, as discussed in the main text.

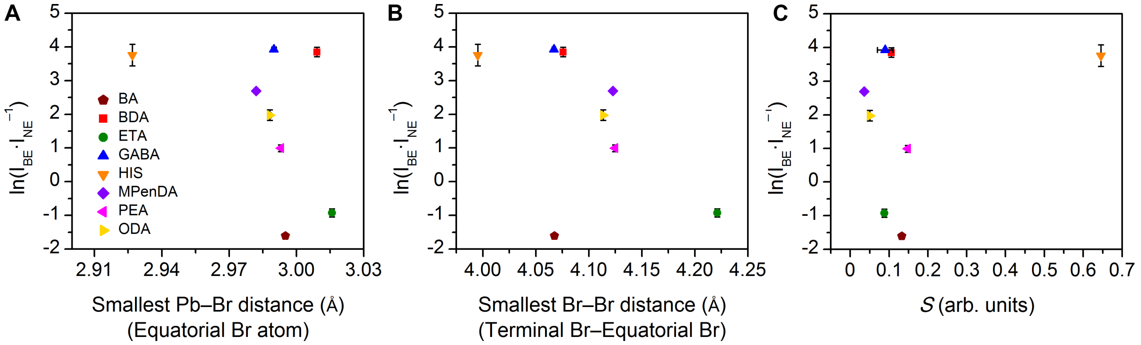

Figure S14. Values of $\ln(I_{BE} \cdot I_{NE}^{-1})$ plotted as a function of (A) the smallest distance between Pb and equatorial Br atoms (within the inorganic layer), (B) the smallest distance between terminal Br and equatorial Br atoms, and (C) octahedral distortion (S). These are representative examples of structural parameters that do not show clear correlation with the broad emission.

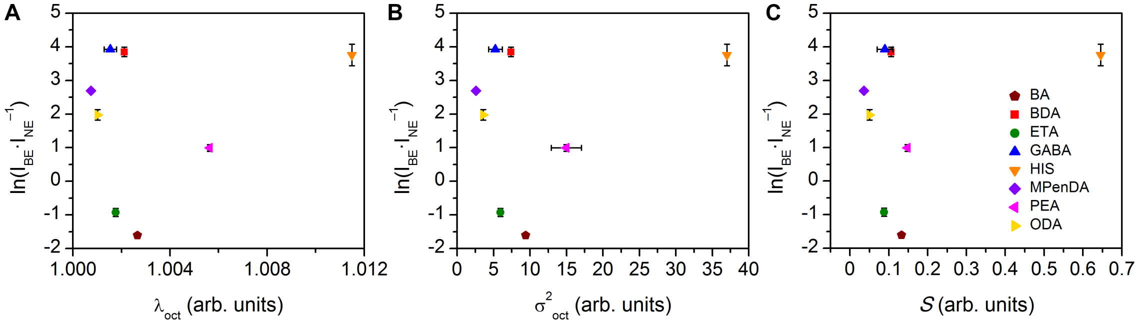

Figure S15. Values of $\ln(I_{BE} \cdot I_{NE}^{-1})$ plotted as a function of (A) λ_{oct}, a measure of octahedral elongation, (B) σ^2_{oct}, the variance of Br–Pb–Br angles within an octahedron, and (C) octahedral distortion (S). Each parameter represents a different way to measure octahedral distortion, though S takes both λ_{oct} and σ^2_{oct} into account and is thus more comprehensive. In each case, no correlation is observed and these three parameters yield very similar plots.

PbBrAngles_witherror.m Script for Calculating Pb–Br angles

```
%This program calculates the angle between three atoms (Pb1-Br-Pb2)
%and the associated error in that value. It then realizes a plane defined
%by Pb1, Pb2, and Pb3 and calculates the in-plane and out-of-plane
%components of the angle (Pb1-Br-Pb2). Input each atom's Cartesian
%coordinates and the values' associated errors as follows:
%PbBrAngles_witherror(Pb1x,Pb1xe,Pb1y,Pb1ye,Pb1z,Pb1ze,...,Pb3z,Pb3ze),
%where Pb1x is the x-coordinate (in Angstroms) of Pb1 and Pb1xe is the
%associated error in that value. Output will be a csv file containing
%D_tilt (i.e., 180-theta), D_in (in-plane component), D_out (out-of-plane)
%and their associated errors. It should be noted that errors will be higher
%if one considers an inherent error in defining the plane from the three
%lead atoms. To remove this error and define all other errors in reference
%to the plane of Pb atoms itself, comment out lines 38-40 and 88-90.
%Written by Adam Jaffe 2/2/16 with the help of Matt Smith

%%
function [] =
PbBrAngles_witherror(Pb1x,Pb1xe,Pb1y,Pb1ye,Pb1z,Pb1ze,Pb2x,Pb2xe,Pb2y,Pb2ye,Pb2z,Pb2ze,Brx,Brxe,B
ry,Brye,Brz,Brze,Pb3x,Pb3xe,Pb3y,Pb3ye,Pb3z,Pb3ze)
Pb1 = [Pb1x;Pb1y;Pb1z];  %Pb1 "point" (vector)
Pb1e = [Pb1xe;Pb1ye;Pb1ze];  %Pb1 error "point" (vector)

Pb2 = [Pb2x;Pb2y;Pb2z];  %Pb2 "point" (vector)
Pb2e = [Pb2xe;Pb2ye;Pb2ze];  %Pb2 error "point" (vector)

Pb3 = [Pb3x;Pb3y;Pb3z];  %Pb3 "point" (vector)
Pb3e = [Pb3xe;Pb3ye;Pb3ze];  %Pb3 error "point" (vector)

Br = [Brx;Bry;Brz];  %Br "point" (vector)
Bre = [Brxe;Brye;Brze];  %Br "point" (vector)

Pb1_Pb2 = Pb1 - Pb2;  %vector between Pb1 and Pb2
Ae = Pb1e + Pb2e;  %error vector between Pb1 and Pb2

Pb2_Pb3 = Pb3 - Pb2;  %vector between Pb2 and Pb3
Be = Pb3e + Pb2e;  %error vector between Pb2 and Pb3

PbPlane = cross(Pb1_Pb2,Pb2_Pb3);  %vector normal to Pb plane
PbPlanee = zeros(3,1);  %error vector normal to Pb plane initialized with zeroes
PbPlanee(1) = Ae(2)*abs(Pb2_Pb3(3)) + Be(3)*abs(Pb1_Pb2(2)) + Ae(3)*abs(Pb2_Pb3(2)) +
Be(2)*abs(Pb1_Pb2(3));  %entry 1 for that cross product error
PbPlanee(2) = Ae(1)*abs(Pb2_Pb3(3)) + Be(3)*abs(Pb1_Pb2(1)) + Ae(3)*abs(Pb2_Pb3(1)) +
Be(1)*abs(Pb1_Pb2(3));  %entry 2 for that cross product error
PbPlanee(3) = Ae(1)*abs(Pb2_Pb3(2)) + Be(2)*abs(Pb1_Pb2(1)) + Ae(2)*abs(Pb2_Pb3(1)) +
Be(1)*abs(Pb1_Pb2(2));  %entry 3 for that cross product error

PbPlanenorm = PbPlane/norm(PbPlane);  %normalized normal vector tp Pb plane
Je = 2*abs(PbPlane(1))*PbPlanee(1) + 2*abs(PbPlane(2))*PbPlanee(2) +
2*abs(PbPlane(3))*PbPlanee(3);  %error on PbPlane dotted with itself; NOT A VECTOR
Ke = vpa(0.5,20)*(Je/abs(norm(PbPlane)));  %error on normalization of Pb plane (CONSTANT)
Le = zeros(3,1);  %error vector for PbPlanenorm
Le(1) = abs(1/norm(PbPlane))*(PbPlanee(1) + ((abs(PbPlane(1))*Ke)/(abs(norm(PbPlane)))));
Le(2) = abs(1/norm(PbPlane))*(PbPlanee(2) + ((abs(PbPlane(2))*Ke)/(abs(norm(PbPlane)))));
Le(3) = abs(1/norm(PbPlane))*(PbPlanee(3) + ((abs(PbPlane(3))*Ke)/(abs(norm(PbPlane)))));

Pb1_Br = Br - Pb1;  %vector between Pb1 and Br
Ce = Bre + Pb1e;  %error vector between Pb1 and Br

Pb2_Br = Br - Pb2;  %vector between Pb2 and Br
De = Bre + Pb2e;  %vector between Pb2 and Br

%%
dot1 = dot(Pb1_Br,PbPlanenorm);
BrProj = Br - PbPlanenorm*dot1;  %projection of Br onto Pb plane
dot1e = abs(PbPlanenorm(1))*Ce(1) + abs(Pb1_Br(1))*Le(1) + abs(PbPlanenorm(2))*Ce(2) +
abs(Pb1_Br(2))*Le(2) + abs(PbPlanenorm(3))*Ce(3) + abs(Pb1_Br(3))*Le(3);  %error on dot product
BrProje = zeros(3,1);    %error vector on BrProj
BrProje(1) = Bre(1) + abs(dot1)*Le(1) + abs(PbPlanenorm(1))*dot1e;
```

```
BrProje(2) = Bre(2) + abs(dot1)*Le(2) + abs(PbPlanenorm(2))*dot1e;
BrProje(3) = Bre(3) + abs(dot1)*Le(3) + abs(PbPlanenorm(3))*dot1e;

Pb1_BrProj = BrProj - Pb1;  %vector between Pb1 and projection of Br onto Pb plane
Ee = BrProje + Pb1e;  %error vector between Pb1 and projection of Br onto Pb plane

Pb2_BrProj = BrProj - Pb2;  %vector between Pb2 and projection of Br onto Pb plane
Fe = BrProje + Pb2e;  %error vector between Pb2 and projection of Br onto Pb plane

dot4 = abs(dot(Pb1_BrProj,Pb2_BrProj));
alpha = dot4/(norm(Pb1_BrProj)*norm(Pb2_BrProj));  %taking the angle between the vectors
D_in = (180/pi)*acos(alpha);  %find D_in
dot2e = 2*abs(Pb1_BrProj(1))*Ee(1) + 2*abs(Pb1_BrProj(2))*Ee(2) + 2*abs(Pb1_BrProj(3))*Ee(3);
%error on Pb1_BrProj dotted with itself; NOT A VECTOR
Me = vpa(0.5,20)*(dot2e/abs(norm(Pb1_BrProj)));  %error on normalization of Pb1_BrProj (CONSTANT)

dot3e = 2*abs(Pb2_BrProj(1))*Fe(1) + 2*abs(Pb2_BrProj(2))*Fe(2) + 2*abs(Pb2_BrProj(3))*Fe(3);
%error on Pb2_BrProj dotted with itself; NOT A VECTOR
Ne = vpa(0.5,20)*(dot3e/abs(norm(Pb2_BrProj)));  %error on normalization of Pb2_BrProj (CONSTANT)

dot4e = ((Ee(1)*abs(Pb2_BrProj(1))) + (Fe(1)*abs(Pb1_BrProj(1)))) + ((Ee(2)*abs(Pb2_BrProj(2))) +
(Fe(2)*abs(Pb1_BrProj(2)))) + ((Ee(3)*abs(Pb2_BrProj(3))) + (Fe(3)*abs(Pb1_BrProj(3))));  %error
on dot product of Pb1_BrProj and Pb2_BrProj CONSTANT

Oe = abs(alpha)*((dot4e/abs(dot4)) + (Me/abs(norm(Pb1_BrProj))) + (Ne/abs(norm(Pb2_BrProj))));
%error on alpha
D_ine = (180/pi)*abs(-(1/sqrt(1 - alpha^2)))*Oe;

%%
NormPlane = cross(Pb1_Pb2,PbPlane);  %vector normal to orthogonal plane
NormPlanee = zeros(3,1);  %error vector normal to orthogonal plane initialized with zeroes
NormPlanee(1) = Ae(2)*abs(PbPlane(3)) + PbPlanee(3)*abs(Pb1_Pb2(2)) + Ae(3)*abs(PbPlane(2)) +
PbPlanee(2)*abs(Pb1_Pb2(3));  %entry 1 for that cross product error
NormPlanee(2) = Ae(1)*abs(PbPlane(3)) + PbPlanee(3)*abs(Pb1_Pb2(1)) + Ae(3)*abs(PbPlane(1)) +
PbPlanee(1)*abs(Pb1_Pb2(3));  %entry 2 for that cross product error
NormPlanee(3) = Ae(1)*abs(PbPlane(2)) + PbPlanee(2)*abs(Pb1_Pb2(1)) + Ae(2)*abs(PbPlane(1)) +
PbPlanee(1)*abs(Pb1_Pb2(2));  %entry 3 for that cross product error

NormPlanenorm = NormPlane/norm(NormPlane);  %normalized vector normal to orthogonal plane to Pb
atoms
Pe = 2*abs(NormPlane(1))*NormPlanee(1) + 2*abs(NormPlane(2))*NormPlanee(2) +
2*abs(NormPlane(3))*NormPlanee(3);  %error on NormPlane dotted with itself; NOT A VECTOR
Qe = vpa(0.5,20)*(Pe/abs(norm(NormPlane)));  %error on normalization of Norm plane (CONSTANT)
Re = zeros(3,1);  %error vector for NormPlanenorm
Re(1) = abs(1/norm(NormPlane))*(NormPlanee(1) + ((abs(NormPlane(1))*Qe)/(abs(norm(NormPlane)))));
Re(2) = abs(1/norm(NormPlane))*(NormPlanee(2) + ((abs(NormPlane(2))*Qe)/(abs(norm(NormPlane)))));
Re(3) = abs(1/norm(NormPlane))*(NormPlanee(3) + ((abs(NormPlane(3))*Qe)/(abs(norm(NormPlane)))));

dot5 = dot(Pb1_Br,NormPlanenorm);
BrProj2 = Br - NormPlanenorm*dot5;  %projection of Br onto orthogonal plane
dot5e = abs(NormPlanenorm(1))*Ce(1) + abs(Pb1_Br(1))*Re(1) + abs(NormPlanenorm(2))*Ce(2) +
abs(Pb1_Br(2))*Re(2) + abs(NormPlanenorm(3))*Ce(3) + abs(Pb1_Br(3))*Re(3);  %error on dot product

BrProj2e = zeros(3,1);   %error vector on BrProj
BrProj2e(1) = Bre(1) + abs(dot5)*Re(1) + abs(NormPlanenorm(1))*dot5e;
BrProj2e(2) = Bre(2) + abs(dot5)*Re(2) + abs(NormPlanenorm(2))*dot5e;
BrProj2e(3) = Bre(3) + abs(dot5)*Re(3) + abs(NormPlanenorm(3))*dot5e;

Pb1_BrProj2 = BrProj2 - Pb1;  %vector between Pb1 and projection of Br onto norm plane
Se = BrProj2e + Pb1e;

Pb2_BrProj2 = BrProj2 - Pb2;  %vector between Pb2 and projection of Br onto norm plane
Te = BrProj2e + Pb2e;

dot8 = abs(dot(Pb1_BrProj2,Pb2_BrProj2));
beta = dot8/(norm(Pb1_BrProj2)*norm(Pb2_BrProj2));
D_out = (180/pi)*acos(beta);
dot6e = 2*abs(Pb1_BrProj2(1))*Se(1) + 2*abs(Pb1_BrProj2(2))*Se(2) + 2*abs(Pb1_BrProj2(3))*Se(3);
%error on Pb1_BrProj2 dotted with itself; NOT A VECTOR
Ue = vpa(0.5,20)*(dot6e/abs(norm(Pb1_BrProj2)));  %error on normalization of Pb1_BrProj2
(CONSTANT)
```

```
dot7e = 2*abs(Pb2_BrProj2(1))*Te(1) + 2*abs(Pb2_BrProj2(2))*Te(2) + 2*abs(Pb2_BrProj2(3))*Te(3);
%error on Pb2_BrProj2 dotted with itself; NOT A VECTOR
Ve = vpa(0.5,20)*(dot7e/abs(norm(Pb2_BrProj2)));  %error on normalization of Pb2_BrProj2
(CONSTANT)

dot8e = ((Se(1)*abs(Pb2_BrProj2(1))) + (Te(1)*abs(Pb1_BrProj2(1)))) +
((Se(2)*abs(Pb2_BrProj2(2))) + (Te(2)*abs(Pb1_BrProj2(2)))) + ((Se(3)*abs(Pb2_BrProj2(3))) +
(Te(3)*abs(Pb1_BrProj2(3))));  %error on dot product of Pb1_BrProj2 and Pb2_BrProj2 CONSTANT

We = abs(beta)*((dot8e/abs(dot8)) + (Ue/abs(norm(Pb1_BrProj2))) + (Ve/abs(norm(Pb2_BrProj2))));
%error on beta
D_oute = (180/pi)*abs(-(1/sqrt(1 - beta^2)))*We;
%%
dot11 = abs(dot(Pb1_Br,Pb2_Br));
gamma = dot11/(norm(Pb1_Br)*norm(Pb2_Br));
D_tilt = vpa(180,20) - (180/pi)*acos(dot(Pb1_Br,Pb2_Br)/(norm(Pb1_Br)*norm(Pb2_Br)));

dot9e = 2*abs(Pb1_Br(1))*Ce(1) + 2*abs(Pb1_Br(2))*Ce(2) + 2*abs(Pb1_Br(3))*Ce(3);  %error on
Pb1_Br dotted with itself; NOT A VECTOR
Xe = vpa(0.5,20)*(dot9e/abs(norm(Pb1_Br)));  %error on normalization of Pb1_Br (CONSTANT)

dot10e = 2*abs(Pb2_Br(1))*De(1) + 2*abs(Pb2_Br(2))*De(2) + 2*abs(Pb2_Br(3))*De(3);  %error on
Pb2_Br dotted with itself; NOT A VECTOR
Ye = vpa(0.5,20)*(dot10e/abs(norm(Pb2_Br)));  %error on normalization of Pb2_Br (CONSTANT)

dot11e = ((Ce(1)*abs(Pb2_Br(1))) + (De(1)*abs(Pb1_Br(1)))) + ((Ce(2)*abs(Pb2_Br(2))) +
(De(2)*abs(Pb1_Br(2)))) + ((Ce(3)*abs(Pb2_Br(3))) + (De(3)*abs(Pb1_Br(3))));  %error on dot
product of Pb1_Br and Pb2_Br CONSTANT

Ze = abs(gamma)*((dot11e/abs(dot11)) + (Xe/abs(norm(Pb1_Br))) + (Ye/abs(norm(Pb2_Br))));  %error
on gamma
D_tilte = (180/pi)*abs(-(1/sqrt(1 - beta^2)))*Ze;
%%
filename = 'PbBrAngles_witherror.csv';  %export condensed full data
fid = fopen(filename, 'w');  %open a new csv in write mode
fprintf(fid, '%s,%s,%s,%s,%s,%s\n', 'D_in','D_in error','D_out','D_out error','D_tilt','D_tilt
error');
fprintf(fid, '%d,%d,%d,%d,%d,%d\n',
double(D_in),double(D_ine),double(D_out),double(D_oute),double(D_tilt),double(D_tilte));
fclose(fid);  %close file

end
```

References

1. *SAINT and SADABS*, Bruker AXS Inc.: Madison, Wisconsin, 2007.
2. G. M. Sheldrick, *SHELXL-97, A program for crystal structure refinement*, Göttingen, 1997.
3. G. M. Sheldrick, *Acta Crystallogr., Sect. A: Found. Crystallogr.*, 2008, **64**, 112-122.
4. O. V. Dolomanov, L. J. Bourhis, R. J. Gildea, J. A. K. Howard and H. Puschmann, *J. Appl. Crystallogr.*, 2009, **42**, 339-341.
5. P. Müller, R. Herbst-Irmer, A. L. Spek, T. R. Schneider and M. R. Sawaya, *Crystal Structure Refinement: A Crystallographer's Guide to SHELXL*, Oxford University Press, New York, 2006.
6. T. Ishihara, J. Takahashi and T. Goto, *Phys. Rev. B*, 1990, **42**, 11099-11107.
7. D. B. Straus, S. Hurtado Parra, N. Iotov, J. Gebhardt, A. M. Rappe, J. E. Subotnik, J. M. Kikkawa and C. R. Kagan, *J. Am. Chem. Soc.*, 2016, **138**, 13798-13801.
8. M. Llunell, D. Casanova, J. Cirera, P. Alemany and S. Alvarez, *SHAPE: Program for the Stereochemical Analysis of Molecular Fragments by Means of Continuous Shape Measures and Associated Tools*, Universitat de Barcelona, 2010.
9. D. Casanova, J. Cirera, M. Llunell, P. Alemany, D. Avnir and S. Alvarez, *J. Am. Chem. Soc.*, 2004, **126**, 1755-1763.
10. J. Cirera, E. Ruiz and S. Alvarez, *Chem. - Eur. J.*, 2006, **12**, 3162-3167.
11. M. Pinsky and D. Avnir, *Inorg. Chem.*, 1998, **37**, 5575-5582.
12. K. Robinson, G. V. Gibbs and P. H. Ribbe, *Science*, 1971, **172**, 567-570.
13. M. S. Minsky, S. Watanabe and N. Yamada, *J. Appl. Phys.*, 2002, **91**, 5176-5181.
